# Osteomyelitis: Epidemiology, Classification, Pathophysiology, Clinical Features, Diagnosis, and Management

**DOI:** 10.1002/mco2.70981

**Published:** 2026-09-21

**Authors:** Qingqiang Lei, Liangbin Lin, Peidong Pu, Jie Chen, Xianchao Su, Shubao Liu, Yanli Tong, Hui Yu

**Affiliations:** ^1^ Department of Urology The Affiliated Hospital of Southwest Jiaotong University, The Third People's Hospital of Chengdu Chengdu China; ^2^ Department of Wound Repair and Rehabilitation Medicine, State Key Laboratory of Trauma and Chemical Poisoning, Trauma Center, Research Institute of Surgery Daping Hospital, Army Medical University Chongqing China; ^3^ Medical Research Center The Affiliated Hospital of Southwest Jiaotong University, The Third People's Hospital of Chengdu Chengdu China; ^4^ Obesity and Metabolism Medicine‐Engineering Integration Laboratory, Department of General Surgery The Affiliated Hospital of Southwest Jiaotong University, The Third People's Hospital of Chengdu Chengdu China; ^5^ The Center of Gastrointestinal and Minimally Invasive Surgery, Department of General Surgery The Affiliated Hospital of Southwest Jiaotong University, The Third People's Hospital of Chengdu Chengdu China; ^6^ Centre for Regenerative Medicine and Health, Hong Kong Institute of Science and Innovation Chinese Academy of Sciences Hong Kong China; ^7^ INSERM U1151, CNRS UMR8253, Institut Necker Enfants Malades Université Paris Cité Paris France

**Keywords:** bone destruction, immune evasion, osteomyelitis, *S. aureus*, targeted therapy

## Abstract

Osteomyelitis (OM) is a severe inflammatory bone disease caused by pathogen invasion, representing a major challenge in orthopedic surgery. *Staphylococcus aureus* (*S. aureus*) is the predominant causative pathogen across all OM subtypes, and its increasing antibiotic resistance has further complicated clinical management. Despite advances in antibiotic therapy and surgical debridement, treatment failure and disease recurrence remain unacceptably high. Emerging evidence suggests that these poor outcomes are largely attributed to the intricate host–pathogen interplay within the bone microenvironment, yet the underlying immunological mechanisms remain incompletely understood. This review provides a comprehensive overview of the epidemiological characteristics, classification, clinical features, pathophysiology, and current diagnostic and therapeutic strategies for OM, with a particular focus on host immune dysregulation in disease initiation and chronicity, elucidating the mechanisms underlying immunosuppressive niche formation and persistent bone destruction. We further dissect the multifaceted immune evasion strategies employed by *S. aureus*, including biofilm formation, intracellular persistence, and abscess barrier construction, which collectively drive infection chronicity and progressive bone destruction. By synthesizing recent advances in osteoimmunology, this review identifies critical knowledge gaps and proposes potential targeted immunomodulatory interventions, offering promising avenues to overcome conventional treatment limitations.

## Graphical abstract

The immunosuppressive in OM

*Staphylococcus aureus* employs multiple physical barriers to evade host immunity during skeletal infection, including biofilm formation on implant surfaces and necrotic bone tissues, invasion into the osteocyte lacuno‐canalicular network (OLCN), intracellular persistence within osteoblasts, osteoclasts, and osteocytes, and construction of abscess communities shielded by multilayered dead and viable immune cells, collectively driving infection chronicity and progressive bone destruction.

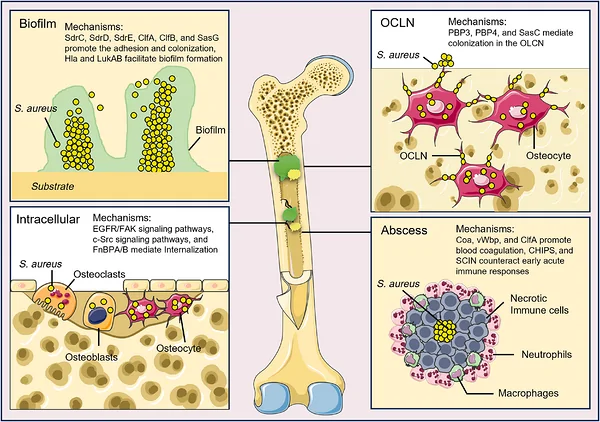

## 1 Introduction

Osteomyelitis (OM), as an inflammatory bone disease, is not uncommon in orthopedic clinic, and its onset is often closely related to open fractures or postoperative implant infection [1, 2]. With its complex pathological process and diverse clinical manifestations, OM has become one of the most challenging infectious diseases in the field of orthopedics, with typical characteristics covering inflammatory response, bone tissue necrosis, dead bone formation, and resistance to antibiotics [3]. These characteristics not only pose a serious threat to the physical health of patients, but also lead to a large number of morbidity and mortality, which has become a major problem faced by orthopedic surgery [4].

The pathogen spectrum of OM shows differential distribution according to the patient's age, anatomical site of the lesion and specific comorbidities. For example, *Staphylococcus aureus* (*S. aureus*) subspecies kingae can be detected in infections of very young children, *Staphylococcus epidermidis* is the main pathogen in patients with implanted medical devices, and post‐traumatic OM may involve *Acinetobacter baumannii* [5, 6, 7]. However, even with the above heterogeneity, *S. aureus* remains the predominant pathogen of all types of OM [8]. This can be attributed to its unique pathogenic capacity, it can easily invade and colonize bone tissue, achieve rapid proliferation through binary fission, then accumulate in large quantities in bone tissue and cause severe bone destruction [9]. In recent years, with the extensive application of antibiotics in clinical treatment, the problem of antibiotic resistance has become increasingly prominent, becoming another major obstacle on the path of OM treatment [10]. Numerous studies have shown that most OM cases are caused by methicillin‐resistant *S. aureus* strains. The emergence of these drug‐resistant strains greatly reduces the efficacy of traditional antibiotic therapy and further increases the difficulty of treatment [11, 12, 13].

The pathogenesis of OM reflects the complex interplay between bacterial virulence traits, antimicrobial immune responses and the physiological functions of bone cells. In the past, studies on OM mainly focused on bacterial biofilm formation, antibiotic permeability and other related aspects. However, with the continuous advancement of research, the academic community has gradually realized that the interaction between the host immune system and pathogens is the key factor determining the infection outcome [14]. A moderate and coordinated immune response can rapidly recognize and eliminate invading pathogens, effectively contain the spread of infection. In contrast, a dysregulated immune response fails to exert its full defensive function, leading to pathogen evasion, subsequent bone destruction and gradual chronic progression of the disease [15, 16]. Persistent *S. aureus* infection can induce feedback mechanisms that suppress immune cell activation, thereby affecting the course of infection [17]. Multiple studies have found that the occurrence and progression of OM are closely correlated with elevated levels of immunosuppressive cells, such as myeloid‐derived suppressor cells (MDSCs) and regulatory T cells (Tregs) [18, 19, 20, 21]. Additionally, in OM patients, the expression of immune activation markers HLA‐DR and CD86 is decreased, while the expression of immune exhaustion markers including T‐cell immunoglobulin mucin‐3 (TIM‐3), programmed cell death protein‐1 (PD‐1) and programmed death‐ligand 1 (PD‐L1) is increased [18]. These changes further demonstrate that the immune system of OM patients is in a dysregulated state, which is unfavorable for pathogen clearance. Therefore, in‐depth research on the abnormal immune mechanisms in OM is expected to open up new avenues for OM treatment and serve as a new breakthrough to tackle this clinical challenge.

This article systematically reviews the epidemiological characteristics, classification, pathophysiology, clinical features, diagnosis, and treatment of OM. Although other pathogens are involved in some subtypes of OM infections, we mainly focus on *S. aureus*, the pathogen that causes the majority of OM cases. In addition, we highlight the critical knowledge gaps regarding immunosuppression and bone destruction in OM, and conduct in‐depth discussions on various host immune evasion strategies adopted by pathogens during the progression of OM, aiming to deepen the understanding of the pathophysiological mechanisms of this important disease. Finally, this paper summarizes the numerous current clinical challenges faced in OM treatment, and proposes potential prevention and treatment strategies targeting these challenges, so as to provide new ideas and directions for the clinical management of OM.

## 2 Epidemiology

### 2.1 Incidence and Prevalence

*S. aureus* is the most prevalent pathogen in OM, particularly in chronic cases, and its immune‐evasive and antibiotic‐resistant properties render it the most destructive [22]. The rising incidence of OM may stem from evolving diagnostic approaches and increasing risk factors such as diabetes [23]. The disease is roughly twice as common in males as in females, with site predilections differing by sex: lower extremities dominate in males, while vertebral infections are slightly more frequent than lower extremity involvement in females [24]. Among monomicrobial infections, *S. aureus* accounts for the vast majority [25]. Smoking is a key modifiable risk factor, with nicotine exacerbating *S. aureus* infection via the Nrf2/Slc7a11 pathway [26]. Marijuana, stimulants, and steroids also significantly increase infection risk [27]. Notably, Black and Hispanic children experience markedly longer hospital stays than white children, suggesting social and healthcare‐access disparities that warrant further investigation [28]. Current evidence quality remains low, particularly regarding the preventive role of long‐term antibiotic therapy against recurrence [25]. Future research should prioritize systematic studies targeting *S. aureus*.

### 2.2 Changes in Epidemiology

Multiple factors influence the epidemiological characteristics of OM. At the pathogen level, the predominant bacterial species have remained relatively stable over time, with the core change being not species replacement but the ongoing evolution of antimicrobial resistance [22, 23]. *S. aureus* consistently ranks first in OM worldwide, and methicillin resistance has become a critical prognostic determinant, identified as an independent risk factor for recurrence after spinal infection surgery [29]. With improved awareness of resistance and standardized antibiotic stewardship, the overall incidence of methicillin‐resistant *S. aureus* OM has declined in recent years [30]. Regarding risk factors, OM onset is directly linked to rising global rates of smoking and intravenous drug abuse, as well as clinical features including thoracic spine involvement, epidural abscess, concurrent bone/joint infection, and bacteremia [29]. At the population level, however, the primary driver of the increasing overall incidence of contemporary OM is the sustained global rise in obesity, diabetes, and peripheral vascular disease [23]. Even in subtypes involving other pathogens, *S. aureus* remains the predominant pathogen across all OM types and must be considered in any empirical antibiotic regimen [31, 32].

## 3 Classification

### 3.1 Classification Based on Disease Course

Traditionally, OM is classified into acute and chronic forms based on the duration of symptoms prior to diagnosis, with the distinction typically drawn at whether symptoms persist beyond 1 month [33]. Acute OM presents with acute inflammatory manifestations, including fever, leukocytosis, lymphadenopathy, and local swelling, often accompanied by cellulitis and trismus, over a course of days to weeks [34, 35]. Bone loss typically requires up to 10 days to become radiographically evident; therefore, negative early imaging findings do not exclude the diagnosis [36]. In contrast, chronic OM generally refers to infections with a prolonged course or specific histopathological features [37]. However, no consensus exists regarding the precise temporal boundary between acute and chronic forms, and histologic changes traditionally considered chronic may also occur in patients with acute onset, making a functional definition of chronic OM difficult to establish and representing a critical issue requiring resolution [38]. Despite ongoing medical advances, OM remains a major clinical challenge, with many patients failing to achieve complete cure, often progressing to chronic or recurrent infection [2, 39], and accompanied by severe complications such as irreversible bone or joint damage, altered skeletal growth dynamics, chronic ulcers, and even amputation, significantly impacting patients' quality of life [40, 41].

### 3.2 Classification Based on Pathogenesis

The complexity of bacterial OM is reflected in numerous classification systems [5, 42], which integrate considerations including infection source, anatomical site, extent of bone involvement, and host physiological and immune status. Based on infection mechanism, OM can be categorized into hematogenous OM, contiguous‐focus OM, post‐traumatic OM, implant‐associated OM, and culture‐negative OM [37]. Different types also exhibit etiological differences. Hematogenous OM is the most common type in children, predominantly affecting long bones and typically occurring at the metaphysis [43]. In adults with hematogenous OM, the vertebral body is the most frequently involved site, attributable to the branching bifurcations of the arterial supply to the vertebral skeleton [44]. Contiguous‐focus OM refers to the spread of infection from adjacent soft tissues to bone. This is the most common type in adults, can occur in any bone, but is most frequently seen in diabetic patients, with its pathogenesis closely associated with neuropathy and peripheral vascular disease [45]. Post‐traumatic OM may be monomicrobial or polymicrobial, depending on the nature of the injury [46]. In terms of causative organisms, polymicrobial OM shares epidemiological similarities with secondary OM in patients with soft tissue wounds [23]. Implant‐associated OM is one of the few dermatologic infection types. Orthopedic implant infections may not initially involve bone, but the infected implant serves as an infection source that subsequently spreads secondarily to bone and progresses to OM [47]. Culture‐negative OM is generally believed to harbor an underlying undetected infection [48]. Potential causes of culture‐negative results include prior antibiotic administration before pathogen isolation and culture, or failure to isolate and detect relatively rare, metabolically quiescent, and slow‐growing pathogens [49, 50].

## 4 Clinical Features

OM can be systematically differentiated by route of infection and disease course, with significant variations in onset patterns, risk factors, and typical presentations across types. The sites of involvement in hematogenous OM are closely correlated with patient demographics. In children, OM predominantly involves the tibial and femoral metaphysis, despite cardinal bone pain and fever, nonspecific presentations often delay diagnosis, necessitating close monitoring and long‐term follow‐up for accurate remission assessment [51]. In adults, hematogenous infection more often involves the vertebrae, with typical manifestations including neck/back pain, local muscle tenderness, and associated fever. Its onset is closely linked to smoking, intravenous drug use, thoracic spine involvement, and bacteremia, among which MRSA infection is an independent risk factor for postoperative recurrence [29]. Nonhematogenous OM usually results from direct spread of adjacent soft tissue infection and is most prevalent in patients with diabetic foot ulcers and chronic wounds. Owing to peripheral vascular disease and local ischemia, clinical manifestations in this group are likewise nonspecific, with pain, edema, and erythema being the common presenting features [52]. Moreover, the combined effect of smoking history and peripheral vascular disease further complicates early diagnosis in these patients [53].

In terms of disease course, acute OM typically has a rapid onset within days and presents with prominent systemic fever; chronic OM, in contrast, rarely displays systemic symptoms, and cutaneous sinuses, chronic nonhealing ulcers, and fracture nonunion are characteristic signs of diagnostic value [33, 54]. Overall, a clear understanding of the distinct features of OM across different routes of infection and stages of disease course constitutes the cornerstone for early recognition, accurate diagnosis, and reduction of poor prognostic outcomes.

## 5 Diagnosis

For patients presenting with acute or progressively worsening musculoskeletal pain accompanied by systemic symptoms such as fever and malaise, the possibility of OM should be considered [54]. However, immunocompromised adults may present with atypical symptoms. Clinical suspicion should be heightened in patients with underlying risk factors, including diabetes, neurovascular disease, chronic ulcers, trauma history, or intravenous drug use [55, 56]. Physical examination findings such as erythema, soft tissue swelling, bony tenderness, or restricted joint motion can increase diagnostic suspicion. A probe‐to‐bone test has some value in ruling out OM in low‐risk diabetic foot patients, and the differential diagnosis should encompass soft tissue infections, gout, fractures, and joint diseases [57]. When clinical uncertainty persists, prompt laboratory and imaging studies should be performed; definitive diagnosis ultimately relies on positive culture results from biopsy of the involved bone tissue [54, 58].

### 5.1 Laboratory Evaluation

Initial laboratory evaluation should include a complete blood count, erythrocyte sedimentation rate, C‐reactive protein, and blood cultures. Leukocytosis may be present in the acute phase but can be absent in chronic infection [59]. Thrombocytosis has some predictive value for OM in patients with chronic leg ulcers [60]. Serial monitoring of inflammatory markers helps assess clinical trends [61]. If blood cultures are positive and X‐ray shows OM changes, more invasive bone biopsy may be avoided [62]. However, it should be noted that elevated markers are only supportive and not diagnostic. For suspected OM, deep bone or soft tissue specimens should be prioritized for culture [56]. Polymerase chain reaction (PCR) has adjunctive value in rapid pathogen identification, particularly in patients who have already received antibiotics [63].

### 5.2 Imaging Examination

The imaging diagnosis of OM often requires a comprehensive approach integrating multiple modalities. Conventional X‐ray remains the first‐line examination, allowing preliminary assessment of bone and soft tissue anatomy and pathological changes [64]. Ultrasonography serves as a complementary tool, helpful in detecting soft tissue fluid collections, periosteal abnormalities, and cortical destruction, while also providing guidance for puncture drainage or biopsy [65]. CT has advantages in demonstrating early bone erosion, foreign bodies, or intraosseous gas, though its overall sensitivity is inferior to other methods [66]. MRI is currently recognized as the most sensitive and specific modality, clearly delineating the extent of infection and involvement of surrounding soft tissues, and can be preferred in unifocal uncomplicated cases [67]. Nuclear medicine techniques such as SPECT and PET are applicable for multifocal lesions or when MRI is contraindicated, and offer high accuracy in chronic OM and distinguishing soft tissue infection [68]. Despite the respective advantages of these methods, existing evidence does not clearly support the universal priority of any single technique across all scenarios, and imaging diagnosis still carries considerable uncertainty. Some studies suggest that combined sequential strategies (e.g., MRI and PET) show promise, but large‐scale prospective studies are needed for validation [69].

## 6 Pathophysiology

The pathophysiology of OM is characterized by a complex interplay between *S. aureus* virulence and host immune responses, driving persistent infection and progressive bone destruction. This section delineates three interconnected pillars underlying OM chronicity. First, physical barriers (including biofilms, osteocyte‐lacuno canalicular network [OLCN] invasion, intracellular persistence, and abscess formation) construct a multilayered defense system that shields the pathogen from immune clearance and antibiotics. Second, *S. aureus* orchestrates profound immunosuppression by directly targeting multiple immune cell populations, from neutrophils and macrophages to T and B cells, establishing a comprehensive inhibitory network across innate and adaptive immunity. Third, bone homeostasis is disrupted as infection promotes osteoclastogenesis, inhibits osteogenic differentiation, and induces osteoblast apoptosis and inflammation, while bone cells also exert immunomodulatory functions.

### 6.1 Formation of Physical Barriers

**FIGURE 1:**
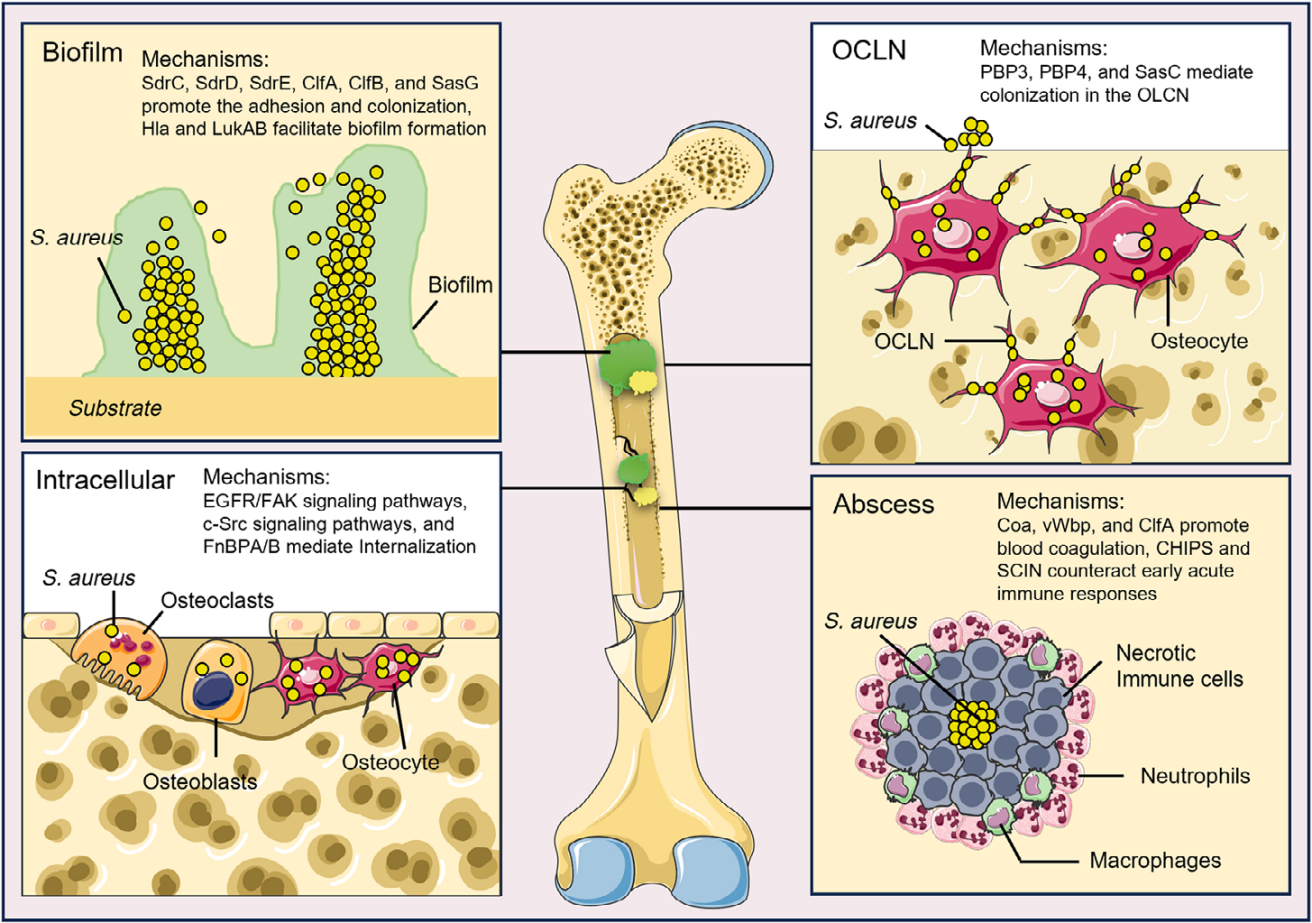
The physical barriers in OM. *S. aureus* employs multiple physical barriers to achieve immune evasion during the process of skeletal infection. First, *S. aureus* utilizes adhesion molecules such as serine aspartate repeat containing protein C (SdrC), SdrD, SdrE, clumping factor A (ClfA), ClfB, and surface protein G (SasG) to promote adhesion and colonization on implant surfaces and necrotic bone tissues, while simultaneously promoting biofilm formation via alpha‐toxin (Hla) and leukocidin AB (LukAB) to resist immune clearance and antibiotic killing. Second, *S. aureus* invades osteocyte‐lacuno canalicular network (OLCN) through penicillin‐binding protein (PBP3), PBP4, SasC, and morphological changes, and persists within osteoblasts, osteoclasts, and osteocytes via the EGFR/FAK signaling pathway, c‐Src signaling pathway, and Fibronectin‐binding protein A/B (FnBPA/B). Furthermore, the bacterium synergistically promotes blood coagulation through Coagulase (Coa), von Willebrand factor‐binding protein (vWbp), and (ClfA), and suppresses early acute immune responses via chemotaxis inhibitory protein of *S. aureus* (CHIPS) and staphylococcal complement inhibitor (SCIN), facilitating the development of abscess communities in the bone marrow cavity and adjacent soft tissues. Within these abscesses, the bacteria are sequestered in the core region, surrounded by multilayers of both dead and viable immune cells, forming a physical barrier that further shields the pathogen from host immune attack.

The chronicity of *S. aureus* is linked to bacterial phenotypic adaptation in vivo, and its physical barrier is critical for evading immune responses and antibiotics [70]. In OM, bacterial colonization and biofilm formation are typically followed by OLCN invasion and intracellular persistence, ultimately leading to dead bones and abscesses; however, these four mechanisms often overlap during chronicity without a strict order [71, 72]. These barriers form a multilayered three‐dimensional defense system that underpins immune evasion and persistent infection (Figure 1). Given these complex barriers, antibiotic therapy alone is almost inevitably ineffective. Surgical debridement is the cornerstone of treatment, aiming to physically remove these obstacles (including sequestra, abscesses, and biofilms). Only after these bacterial fortresses are eliminated can antibiotics and the immune system take effect.

#### 6.1.1 Formation of Biofilm

Biofilms are bacterial communities encased in a matrix of polysaccharides, eDNA, and proteins, creating a microenvironment that promotes bacterial adaptation [73]. In biofilms, *S. aureus* shows increased tolerance to nutrient deprivation and antibiotics [72, 74], while also blocking macrophage and neutrophil infiltration [75]. Strong biofilm‐forming and multidrug‐resistant strains often carry specific genetic variants [76]. Beyond serving as a physical barrier, biofilms evade immune killing by inactivating effector molecules and promoting anti‐inflammatory phenotypes [77], driving metabolic and functional changes in immune cells that lead to dysregulated responses and persistent infections [78]. Moreover, higher infection doses may accelerate biofilm formation and alter host immune responses, complicating elucidation of underlying mechanisms [79].

Biofilm formation occurs in two sequential steps: adhesion to a solid matrix, followed by cell‐to‐cell aggregation [80]. Adhesion to host tissues is an initial key event in most infections [81, 82], and intercellular adhesion sites in *S. aureus* are essential for biofilm formation [83]. Surface‐anchored proteins serine aspartate repeat containing protein C (SdrC), SdrD, and SdrE promote adhesion and colonization [84, 85], while other microbial surface components recognizing adhesive matrix molecules (MSCRAMMs; e.g., clumping factor A [ClfA], ClfB, surface protein G [SasG]) facilitate aggregation on prosthetic and biological surfaces [85, 86]. Quantitative mass spectrometry has identified alpha‐toxin (Hla) and leukocidin AB (LukAB) as key biofilm‐secreted molecules that promote biofilm formation and inhibit macrophage phagocytosis in vivo (Figure 1) [87]. Advances in understanding these unique properties have fueled growing optimism for targeted antibiofilm strategies against *S. aureus*.

#### 6.1.2 OLCN Invasion

Osteocytes, the most mature cells of the osteoblast lineage, are embedded in lacunae within the rigid extracellular matrix and extend slender processes through canaliculi that connect adjacent lacunae, forming a large three‐dimensional network known as the osteocyte OLCN [88, 89]. The OLCN serves multiple key functions, including mechanosensation, bone‐remodeling balance, and mineral homeostasis, while also acting as the transport channel for blood, nutrients, and signaling pathways within bone tissue [90]. These features provide *S. aureus* with a favorable niche to survive within the OLCN and evade immune surveillance, potentially explaining the prolonged course of chronic bone infections [91].

Recent studies have confirmed *S. aureus* colonization within the OLCN of OM patients via immunohistochemistry and light microscopy, highlighting its clinical relevance and offering new insights into immune evasion and persistence [92]. In vitro models have also demonstrated bacterial invasion of the OLCN [93], with malformed bacteria entering tubular structures through asymmetric binary fission and migrating via proliferation at the leading edge [94]. Mechanistic studies have identified penicillin‐binding proteins PBP3 and PBP4, as well as the surface adhesion protein SasC, as essential for OLCN colonization (Figure 1) [95, 96, 97]. However, current research has not deeply explored the causes, specific biological processes, or clinical case correlations of OLCN invasion. Therefore, further in‐depth investigation integrating clinical cases of chronic *S. aureus* bone infection represents a promising future research direction.

#### 6.1.3 Intracellular Infection

The role of intracellular infection in OM remains an active area of research. Upon entering host cells, *S. aureus* is shielded by cell membranes from antibiotics, antibodies, and complement, thereby evading immune surveillance, while infected cells may also serve as vectors for bacterial dissemination. In the skeletal environment, *S. aureus* can infect and proliferate within osteoclasts, revealing a unique bacterial reservoir mechanism [98, 99]. Additionally, *S. aureus* invades and persists in osteoblasts and osteocytes, contributing to antibiotic resistance [100, 101, 102]. Long‐term intracellular persistence in osteocytes, driven by their poor intracellular antibacterial responses, underlies infection recurrence and is frequently observed in clinical OM cases [103, 104]. Infected osteoblasts and osteocytes not only act as reservoirs but also disrupt bone homeostasis by promoting osteoclast‐mediated bone resorption [105]. Mechanistically, the EGFR/FAK and c‐Src pathways mediate osteoblast internalization [106], while phagocyte internalization involves Fibronectin‐binding protein A (FnBPA) and Fibronectin‐binding protein B (FnBPB) binding to fibrinogen and connecting to α5β1 integrin on macrophages/neutrophils (Figure 1) [107]. However, current understanding of intracellular infection remains limited, with unclear duration of bacterial survival and extent of cellular involvement. Thus, further investigation using in vivo OM models is needed to clarify its role.

#### 6.1.4 Formation of Dead Bones and Abscesses

*S. aureus* infection disrupts bone blood supply and induces tissue necrosis, forming avascular devitalized sequestra that provide an ideal surface for robust biofilm formation while remaining inaccessible to immune cells and systemic antibiotics [2]. Additionally, *S. aureus* establishes long‐term infection through abscess formation in bone marrow and peri‐implant soft tissues [108]. Abscesses consist of central staphylococcal abscess communities (SACs) encased by fibrin pseudocapsules and infiltrating immune cells, which impede immune cell and antibiotic penetration [109]. Bacterial coagulation is mediated by coagulase (Coa) and von Willebrand factor‐binding protein (vWbp) activating prothrombin to convert fibrinogen to fibrin [110], while ClfA also binds fibrinogen and promotes platelet aggregation via fibrinogen‐ or complement‐dependent mechanisms [110, 111]. Immune evasion is further aided by secreted proteins such as chemotaxis inhibitory protein of *S. aureus* (CHIPS) and staphylococcal complement inhibitor (SCIN) that counteract early acute immune responses (Figure 1) [112]. As bacterial aggregation progresses, extensive immune cell infiltration causes host tissue damage, and immune cell death further generates a protective barrier that blocks new immune cell entry into the abscess [113]. The high internal pressure, limited blood flow, and hypoxic/low‐pH environment within the abscess impair immune function and antibiotic activity [114]. To sustain persistence, *S. aureus* within abscesses utilizes iron‐binding proteins iron‐regulated surface determinant protein A (IsdA), IsdB, and IsdH to capture heme from hemoglobin as an iron source for growth and reproduction [115, 116].

### 6.2 Immunosuppression in OM

**FIGURE 2:**
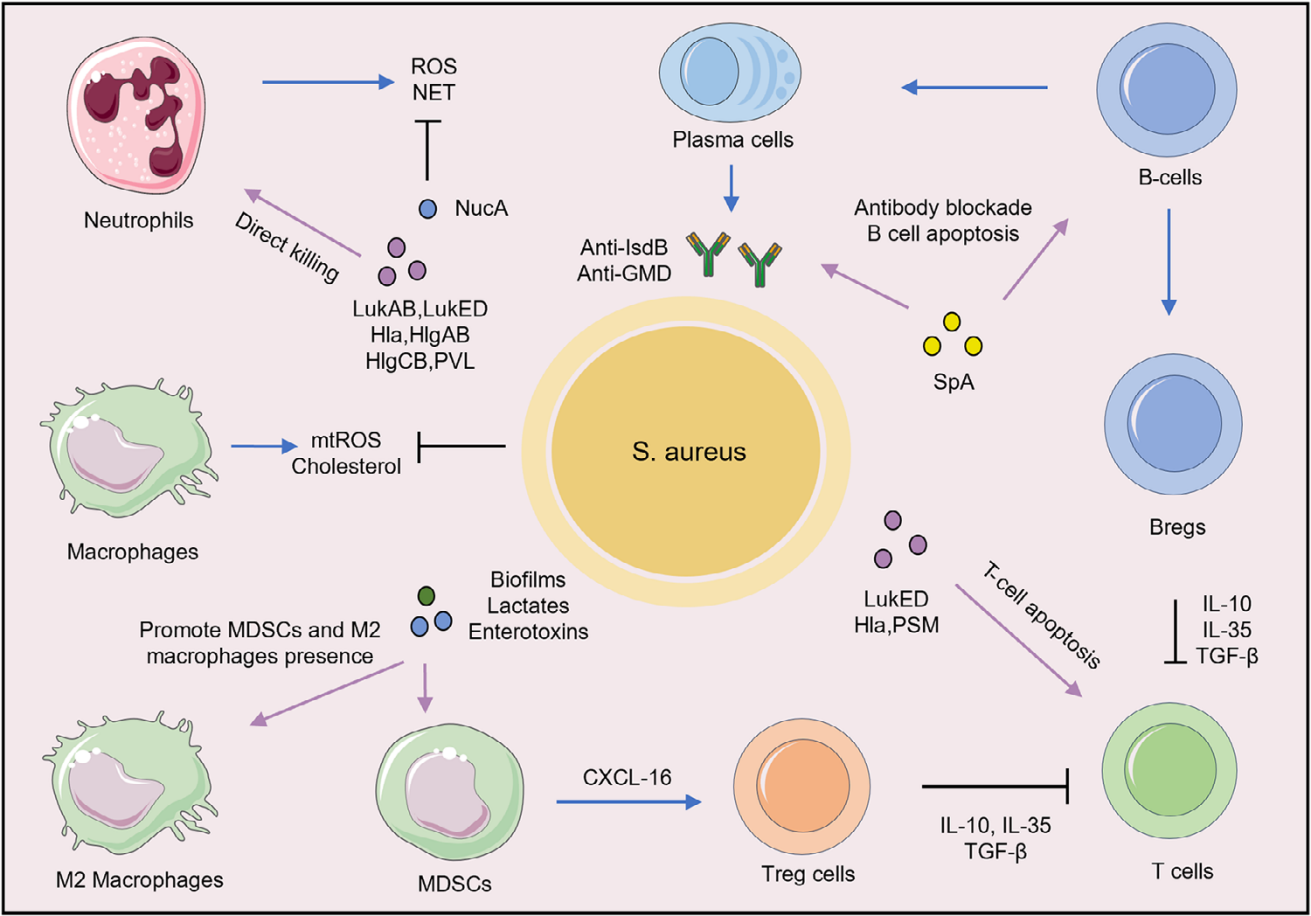
The immunosuppressive microenvironment in OM. *S. aureus* maintains an immunosuppressive microenvironment in OM through multiple pathways. *S. aureus* directly eliminates neutrophils through LukAB, LukED, Hla, Gamma‐hemolysin AB (HlgAB), HlgCB, and panton‐valentine leukocidin (PVL), and inhibits NET formation via NucA. At the same time, it impairs the bactericidal activity of macrophages by suppressing mtROS production and cholesterol metabolism. It neutralizes anti‐IsdB and anti‐GMD antibodies through SpA, which also mediates B‐cell apoptosis. Furthermore, *S. aureus* directly kills T cells via LukED, Hla, and phenol‐soluble modulin (PSM). In addition, it sustains immunosuppression by promoting the generation and recruitment of MDSCs, M2 macrophages, Treg cells, and Breg cells.

In addition to forming the aforementioned physical barriers to achieve immune evasion, *S. aureus* can also directly act on immune cells through various means (such as directly killing immune cells, inhibiting bactericidal capabilities, inducing cell apoptosis, and promoting the generation of immunosuppressive cells), thereby constructing a comprehensive inhibitory network spanning from innate immunity to adaptive immunity (Figure 2). This profound immunosuppression represents the core pathogenic mechanism by which *S. aureus* persists and leads to the chronicity of infections.

#### 6.2.1 Neutrophils

Neutrophils are the primary effectors in early infection, eliminating bacteria via phagocytosis, ROS production, and antimicrobial peptide release. *S. aureus* infection is characterized by early neutrophil infiltration and a dominant inflammatory response [17]. And with a significantly elevated neutrophil‐to‐lymphocyte ratio (NLR) serving as a key predictor of OM [117, 118]. NLR combined with CRP, TNF‐α, and IL‐6 shows improved diagnostic accuracy for clinical management [118]. A new study reveals that during bone marrow infection, Mrgpra2^+^ neutrophils drive ROS‐dependent neutrophil extracellular trap (NET) release via Mrgpra2 and TNFR signals converging on a PLC/Ca^2^^+^/PKC/NADPH oxidase axis [119]. The thermonuclease NucA of *S. aureus* degrades extracellular DNA/RNA, allowing the pathogen to escape NETs [120]. The *S. aureus* cytotoxin LukAB kills human neutrophils by targeting the CD11b subunit of integrin Mac‐1 via a glutamic acid residue at position 323 of its C‐terminus [121, 122]. Genetic background also significantly influences neutrophil function and activation, thereby affecting OM onset and progression [123]. LukED, a pore‐forming toxin, plays a critical role in immune evasion and nutrient acquisition [124], and has recently been shown to bind and kill neutrophils by targeting chemokine receptors CXCR1 and CXCR2 in a specific and saturable manner [125]. Additionally, *S. aureus* secretes other pore‐forming toxins (e.g., Hla, Gamma‐hemolysin AB [HlgAB], HlgCB, and panton‐valentine leukocidin [PVL]) that directly disrupt neutrophil membranes (Figure 2) [126, 127]. These findings highlight the dual role of neutrophils in OM, essential for bacterial clearance yet capable of driving tissue damage when overactivated. *S. aureus* evades host defense through an array of toxins that specifically target neutrophils, suggesting that therapeutic strategies should focus on preserving neutrophil function while neutralizing virulence factors, rather than simply enhancing or suppressing their activity.

#### 6.2.2 Macrophages

**FIGURE 3:**
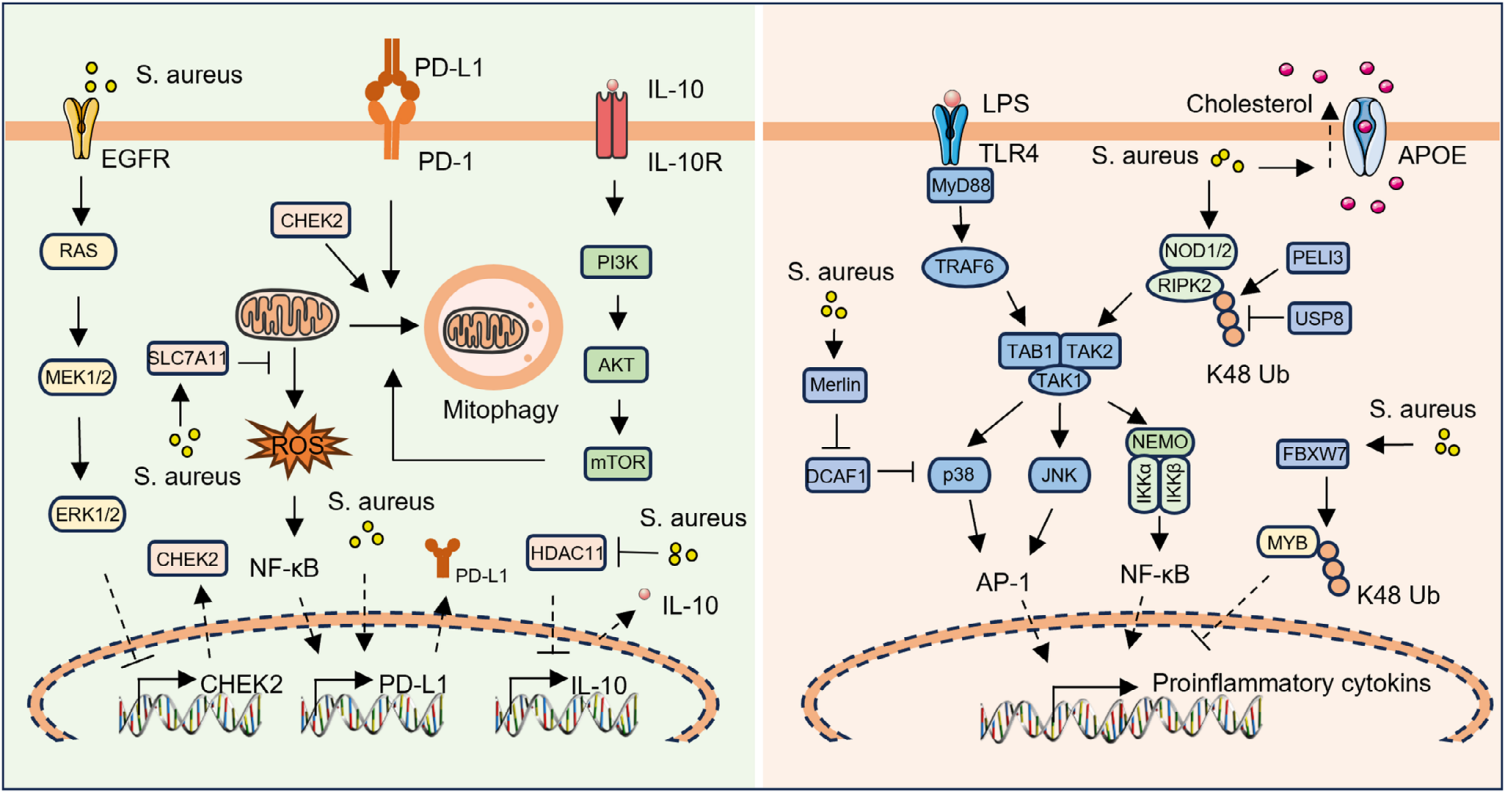
Regulation of macrophage function by *S. aureus*. *S. aureus* exacerbates the progression of OM by inhibiting the bactericidal capacity of macrophages and promoting the expression of inflammatory factors. *S. aureus* inhibits CHEK2 expression via the EGFR/MEK1/2/ERK1/2 pathway, thereby reducing mitochondrial ROS (mtROS) production; upregulates programmed cell death protein‐1 (PD‐1) and programmed death‐ligand 1 (PD‐L1) in macrophages to activate mitophagy and suppress mtROS; and downregulates intracellular mtROS by promoting SLC7A11 expression, while SLC7A11 blockade enhances PD‐L1 expression through the ROS‐NF‐κB axis. IL‐10 promotes mitophagy and mtROS clearance via the mTOR pathway, and *S. aureus* promotes IL‐10 production by inhibiting HDAC11. Additionally, *S. aureus* enhances LPS‐induced downstream signaling by promoting Merlin expression to inhibit DCAF1 activity, and activates the NOD/RIPK2 pathway to promote inflammatory cytokine production. PELI3 inhibits RIPK2‐mediated signaling through K48‐linked ubiquitination, whereas USP8 promotes inflammatory cytokine production by removing this modification. FBXW7 promotes IL‐6 and IL‐23a expression via K48‐linked ubiquitination of MYB. Furthermore, *S. aureus* impairs macrophage bactericidal capacity by promoting cholesterol efflux through apolipoprotein E (ApoE).

*S. aureus* survival within macrophages is critical for immune evasion and promotes OM pathogenesis (Figure 3). In a murine OM model, *S. aureus* suppresses CHEK2 expression via the EGFR/MEK1/2/ERK1/2 pathway, inducing excessive mitophagy and reduced mitochondrial ROS (mtROS), thereby impairing macrophage bactericidal function and driving uncontrolled bone resorption [128, 129]. Pharmacological blockade of EGFR or MEK1/2 restored CHEK2 expression, enhanced bacterial clearance, and improved bone microstructure, highlighting EGFR‐MEK1/2 as a metabolic immune checkpoint and suggesting that combining pathway inhibition with antibiotics represents a promising therapeutic strategy [128, 129]. Additionally, PD‐1/PD‐L1 is upregulated in macrophages surrounding bone abscesses and in OM patient bone marrow, activating mitophagy and suppressing mtROS production to compromise macrophage killing, while neutralizing antibodies against PD‐L1 or PD‐1 reduced mitophagy, enhanced bacterial clearance, and mitigated bone destruction [130]. Similarly, SLC7A11 is significantly upregulated in macrophages of *S. aureus* OM mice; its inhibition or knockout promoted bactericidal function, reduced bacterial load, and improved skeletal structure by lowering mtROS levels, and blocking SLC7A11 upregulates PD‐L1 via the ROS‐NF‐κB axis, with combined SLC7A11/PD‐L1 targeting significantly improving bacterial clearance, suggesting a promising dual‐targeted therapy [131]. Moreover, IL‐10 promotes mitophagy and mtROS clearance via the mTOR pathway to enhance intracellular *S. aureus* survival, and HDAC11, a transcriptional repressor of IL‐10, is inhibited during infection, leading to increased IL‐10 production [132]. Collectively, these findings underscore that *S. aureus* exploits multiple macrophage immune checkpoints to subvert host defenses via mitochondrial dysfunction, and that targeting these pathways holds great promise for overcoming immune evasion and improving outcomes in chronic OM.

The inflammatory microenvironment around abscesses plays a key role in *S. aureus* OM progression [133]. Beyond impairing macrophage mtROS production for immune evasion, *S. aureus* disrupts bone homeostasis by promoting inflammatory cytokine production in macrophages (Figure 3). Merlin, a cytoskeletal scaffold protein encoded by NF2, is significantly elevated in macrophages from OM patients and promotes inflammatory responses by inhibiting DCAF1 activity, thereby exacerbating OM [134, 135]; DCAF1 deletion conversely promotes inflammatory cytokine production via p38 overactivation [136]. FBXW7, an E3 ubiquitin ligase, is also upregulated in OM macrophages and promotes IL‐6, IL‐23a, and NOS2 expression by mediating K48‐ubiquitination of MYB, thereby driving OM progression [137, 138]. RIPK2 transmits NOD1/2 downstream signals to activate NF‐κB and MAPK pathways, promoting proinflammatory cytokine transcription [139]. Previous studies have reported that bacteria can promote the development of OM by activating the NOD/RIPK2 pathway [140]. The latest study found that E3 ubiquitin ligase PELI3 targets the degradation of RIPK2 by mediating the K48 ubiquitination modification of RIPK2 in macrophages, inhibiting the occurrence of *S. aureus*‐induced OM [141]. However, USP8 promotes NF‐κB activation and the production of inflammatory cytokines by removing K48 ubiquitination of RIPK2, thereby facilitating the development of *S. aureus*‐induced OM [142]. Collectively, these findings reveal that *S. aureus* exploits multiple macrophages signaling regulators to fuel inflammatory bone destruction, and targeting these inflammatory checkpoints may offer novel therapeutic strategies for OM.

Changes in cholesterol content in macrophages can affect their bactericidal ability [143]. The results found that, *S. aureus* induces cholesterol loss in BMDMs in OM mice [144]. Apolipoprotein E (ApoE) is a 35 kDa glycoprotein that belongs to a class of cellular proteins involved in lipid metabolism and plays an important role in cholesterol efflux and reversal of cholesterol transport [145]. Further research found that ApoE deletion enhances the bactericidal capacity of macrophages by promoting cholesterol biosynthesis, thereby attenuating *S. aureus* OM infection (Figure 3) [144]. The study elucidated this phenomenon from the perspective of ApoE‐mediated metabolic reprogramming, suggesting that reprogramming of cholesterol metabolism serves as a critical host defense strategy against *S. aureus* OM. Therefore, in the treatment of *S. aureus* OM, cholesterol metabolism can be considered as a potential therapeutic target.

#### 6.2.3 MDSCs

MDSCs are a heterogeneous group of immature monocytes and granulocytes that expand during cancer, inflammation, and infection, and have the ability to significantly inhibit the activation and function of T cells and macrophages [146, 147]. Based on the expression and function of surface markers, bone MDSCs can be broadly divided into two groups: single‐cell myeloid‐derived suppressor cells (M‐MDSCs) and granular myeloid‐derived suppressor cells (G‐MDSCs) [148, 149]. MDSCs also play a key role in bacterial infections [150, 151, 152].

Increased MDSCs in *S. aureus*‐infected mice may establish an immunosuppressive milieu that perpetuates chronic infection, thereby markedly elevating infection burdens [21, 153, 154]. During *S. aureus* biofilm‐associated otitis media, CD11b^+^ Gr‐1^+^ MDSCs represent the predominant infiltrating immune cells [155], with further characterization identifying CD11bʰ^i^ Ly6G^+^ Ly6C^+^ as markers of G‐MDSCs, confirmed by typical nuclear morphology in cytospin preparations [156]. Recent work in prosthetic joint infection (PJI) has uncovered a previously unrecognized Ly6G^+^ Ly6C^+^ F4/80^+^ MHCII^+^ MDSC subset that exhibits in vitro immunosuppressive properties akin to G‐MDSCs, yet shows significantly elevated expression in cytokine responses, inflammatory cell death, and monocyte differentiation pathways [157], indicating that F4/80^+^ MHCII^+^ MDSCs possess an underappreciated phenotypic plasticity and can transition from granulocytic to monocytic lineages during biofilm infection. MDSCs exert immunosuppression largely through IL‐10 production, which inhibits macrophage and T‐cell functions, thus facilitating persistent *S. aureus* colonization [155, 158]. In both PJI patients and mouse models, local chemokines CXCL1 and CXCL2 are markedly upregulated, promoting MDSC recruitment to infection sites [159, 160], while IL‐12 is also significantly increased and further drives MDSC recruitment and proliferation [160]. Single‐cell RNA sequencing has revealed that glycolytic and HIF‐1α pathways are strongly enriched in G‐MDSCs from PJI patients, and genetic or pharmacological interference with these pathways in murine PJI models alleviates G‐MDSC‐mediated immunosuppression and reduces bacterial loads [161], underscoring the critical role of the glycolysis/HIF1α axis in sustaining G‐MDSC anti‐inflammatory activity and biofilm persistence.

The results found that *S. aureus* biofilms can induce the amplification, activation, and polarization of MDSCs both in vivo and in vitro [162]. *S. aureus* biofilm upregulates the fetuin‐like modification of MDSCs and M2 macrophages in a dose‐dependent manner, thereby promoting the proliferation of MDSCs and M2 macrophages [163]. ATP synthase in *S. aureus* biofilm shifts the host immune response toward anti‐inflammatory by increasing the recruitment of MDSCs, which attenuate the proinflammatory activity of macrophages, leading to chronic infection [164]. The synovial fluid of patients with PJI had higher levels of D‐lactic acid and IL‐10 than those in the control group [165]. Further studies have found that *S. aureus*‐derived lactate inhibits histone deacetylase 11 (HDAC11) and increase histone 3 acetylation at the Il‐10 promoter, thereby enhancing Il‐10 transcription in MDSCs and macrophages [165]. Additionally, studies have reported that staphylococcal enterotoxins are important regulators of MDSC generation, suggesting that staphylococcal enterotoxins may become novel therapeutic targets in *S. aureus* infectious diseases (Figure 2) [166].

#### 6.2.4 T Cells

T cells and their subsets are critical for host defense against *S. aureus* in OM [167]. In humanized mice, *S. aureus* infection increases human T‐cell numbers and promotes their clustering around SACs, indicating effective activation and proliferation in infected bone [168]. Chemokines CCR2 and CCR6 enhance late‐stage host defense by facilitating T‐cell infiltration into bone marrow and draining lymph nodes [169, 170]. However, during persistent infection, T cells upregulate inhibitory receptors (LAG‐3, TIM‐3) and exhaustion markers, alongside inhibitory cytokines and feedback loops, which collectively reduce T‐cell proliferation and tissue infiltration [17]. LAG‐3 and TIM‐3 are important coinhibitory receptors for T‐cell activation and play an important role in regulating T‐cell responses [171, 172]. Indeed, OM patients show increased LAG‐3^+^ CD4^+^ and CD8^+^ T cells in peripheral blood with impaired proliferation and IFN‐γ secretion [173], and in a *S. aureus* PJI mouse model, multiomics revealed a terminally exhausted Th1/Th17 mixed population with elevated LAG‐3/TIM‐3 and diminished proinflammatory cytokines (IFN‐γ, IL‐2, TNF‐α, IL‐17) [174]. Thus, T‐cell exhaustion markers may serve as functional biomarkers for OM outcomes, and checkpoint blockade could offer therapeutic potential. Additionally, *S. aureus* secretes pore‐forming toxins (e.g., LukED, Hla, phenol‐soluble modulin [PSM]) that disrupt T‐cell differentiation, activation, and induce apoptosis, facilitating immune evasion (Figure 2) [125, 175].

Tregs are essential for maintaining peripheral tolerance, preventing autoimmune diseases such as Type 1 diabetes, and limiting chronic inflammatory conditions like asthma and inflammatory bowel disease [176, 177, 178, 179]. Recent years have seen significant advances in understanding the molecular mechanisms underlying Treg‐mediated inhibition [180]. In OM, Tregs suppress T‐cell responses against *S. aureus*, thereby promoting disease progression. Notably, the proportion of CD4^+^ CD25^+^ Foxp3^+^ Tregs in the blood of OM patients is significantly higher than in healthy controls [22, 23], suggesting that Tregs may inhibit bacterial clearance by T cells, thus facilitating bacterial persistence. In OM mice, IL‐2 expression and the proportion of peripheral CD4^+^ CD25^+^ Foxp3^+^ Tregs were markedly elevated, along with increased mRNA expression of Foxp3, CTLA‐4, and p‐STAT5 protein in Tregs [181], implicating the IL‐2/STAT5 pathway in OM pathogenesis. Excessive immunosuppression may sustain chronic infectious inflammation, representing a potential therapeutic target. Serum CXCL4 levels were significantly elevated in chronic OM patients, and Treg proportions decreased upon STAT5 inhibition [182]. Single‐cell RNA sequencing of synovium from PJI patients revealed expansion and enhanced activity of M‐MDSC Treg cells, with marked upregulation of CXCL16 in M‐MDSCs and corresponding increases in its receptor CXCR6 on Tregs [183]. Mechanistically, M‐MDSCs recruit and activate Tregs via CXCL16–CXCR6 interactions, while Tregs secrete TGF‐β to promote M‐MDSC proliferation and immunosuppressive function (Figure 2) [183]. Highlight the critical role of CXCR6 in shaping the immunosuppressive microenvironment and biofilm persistence in PJI, and offering a potential therapeutic target.

#### 6.2.5 B Cells

B cells are key adaptive immune cells that primarily mediate humoral immunity [184, 185]. In OM patients, serum antibody testing against *S. aureus* shows significantly elevated IgG titers with variable patterns, correlating with prognosis [186, 187]. However, some patients harbor pathogenic antibodies targeting the heme‐scavenging protein IsdB, which is associated with poor outcomes [188, 189]. Glucosaminidase (GMD), a critical enzyme in *S. aureus* cell wall peptidoglycan synthesis [190], has been identified as a potential serum biomarker for *S. aureus* infection, and deficiency in circulating anti‐GMD antibodies is linked to serious adverse outcomes [191, 192]. Though further prospective studies are needed to clarify its immune role in OM. Both neutralizing and non‐neutralizing anti‐GMD antibodies exist in patients, and screening may help identify candidates for passive immunization prior to surgery [193, 194]. Mechanistically, anti‐GMD monoclonal antibodies confer passive immunity by recruiting macrophage infiltration into SACs and giant clusters, followed by direct phagocytosis of *S. aureus* [195, 196]. Collectively, these findings highlight the potential of humoral immune responses against *S. aureus*, and measuring host antibody responses may serve as a predictor of persistent infection with prognostic value.

However, during persistent infection, *S. aureus* is capable of regulating B‐cell survival and function, as well as influencing humoral antibody responses. *S. aureus* secretes staphylococcal protein A (SpA), which binds to the Fcγ and Fab domains of specific antibodies, thereby blocking antibody‐mediated phagocytosis [197, 198]. Meanwhile, SpA can also initiate proliferative apoptosis in B cells (Figure 2) [199, 200]. Additionally, *S. aureus* staphylokinase (Sak) strongly activates human plasminogen on the cell surface, leading to the cleavage and degradation of IgG [201]. Regulatory B cells (Bregs) are a subset of B cells with immunosuppressive functions. They inhibit the proliferation and function of effector T cells and other immune cells by producing IL‐10, IL‐35, and TGF‐β [202, 203]. The results found that Bregs and memory B cells were significantly increased in OM patients [18]. Breg cells interact with MDSCs and Tregs to maintain an immunosuppressive microenvironment in OM. The persistent augmentation of immunosuppressive cells is one of the key factors contributing to the chronicity of infection, poor antibiotic efficacy, and high recurrence rates, which may consequently impact the skeleton by directly infecting osteocytes.

### 6.3 Bone Homeostasis in OM

**FIGURE 4:**
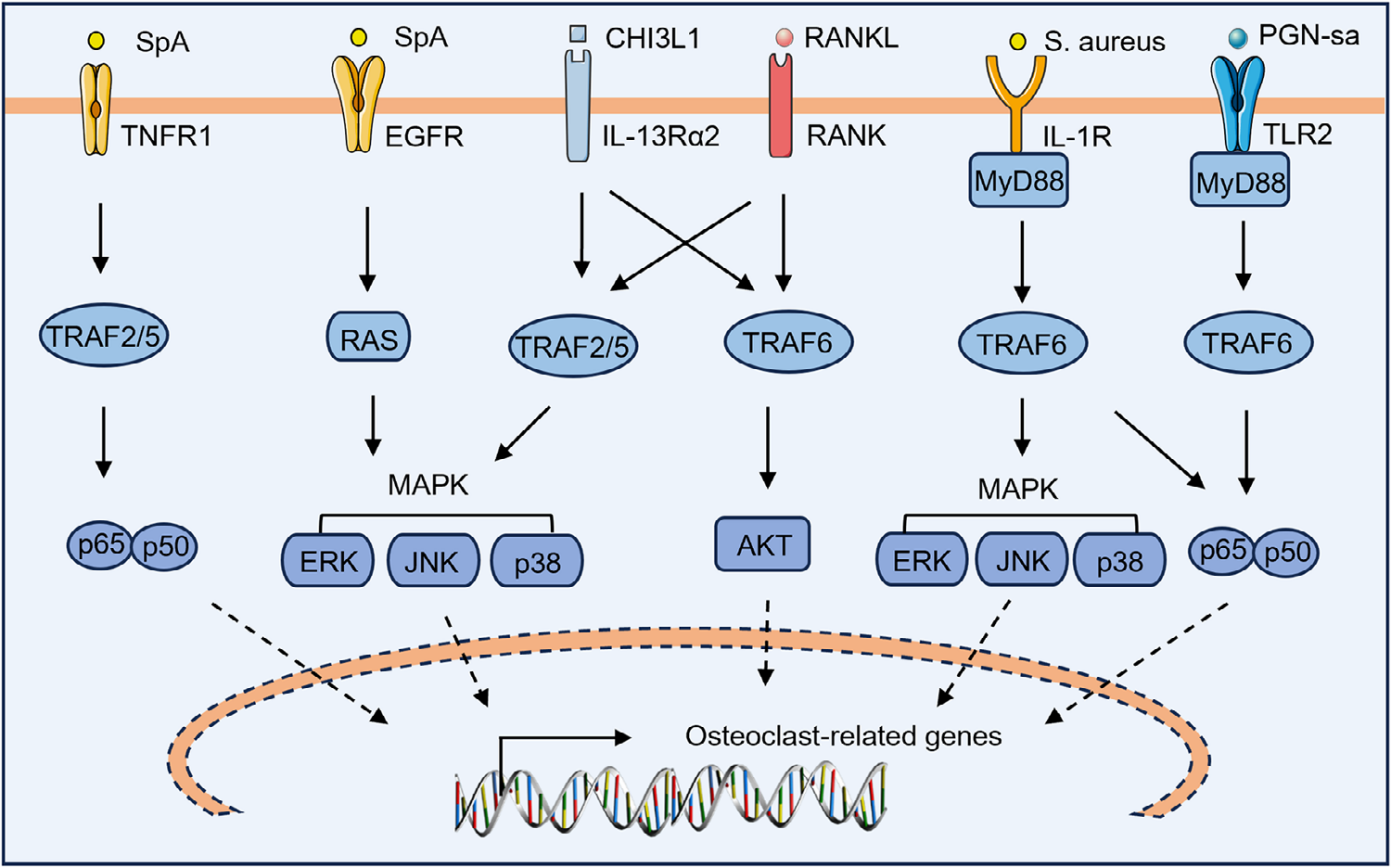
*S. aureus* promotes osteoclastogenesis. *S. aureus* can promote osteoclastogenesis by activating multiple signaling pathways. SpA activates the NF‐κB and MAPK signaling pathways via TNFR1 and EGFR, thereby inducing osteoclast formation. *S. aureus* can upregulate the expression of Chitinase 3‐like 1 (CHI3L1), which binds to its receptor IL‐13Rα2, enhancing the activation of MAPK and AKT pathways induced by RANKL and promoting osteoclast differentiation. *S. aureus* can also drive osteoclastogenesis through the IL‐1R/MyD88 signaling pathway. By secreting *S. aureus* peptidoglycan (PGN‐sa), *S. aureus* mediates the TLR2/NF‐κB/NFATc1 signaling pathway to promote osteoclast formation, thereby facilitating the progression of OM.

**FIGURE 5:**
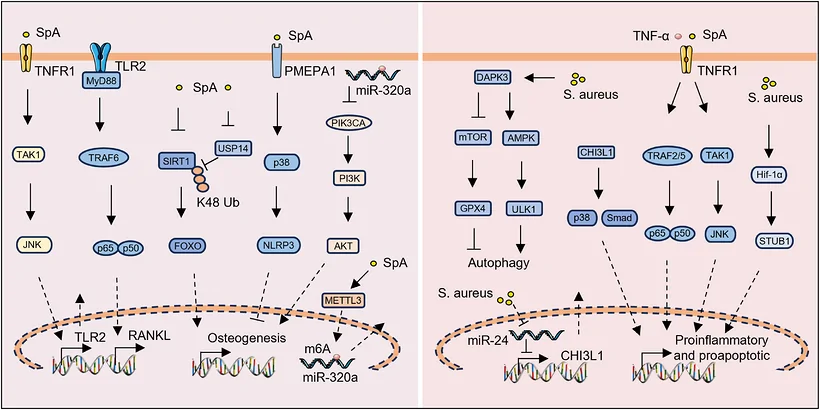
*S. aureus* inhibits osteogenic differentiation. *S. aureus* can exacerbate OM by inhibiting osteogenic differentiation and promoting the expression of inflammatory factors that induce osteoblast apoptosis. *S. aureus* SpA promotes TLR2 expression in osteoblasts via the TNFR1/JNK pathway, and TLR2 downstream signaling enhances RANKL production. SIRT1 activates the FOXO pathway to promote bone formation; USP14 stabilizes SIRT1 through deubiquitination, whereas SpA downregulates both SIRT1 and USP14. SpA induces PMEPA1 upregulation and inhibits osteogenic differentiation via the p38MAPK/NLRP3 axis, and also upregulates METTL3, whose m6A modification promotes miR‐320a expression, which suppresses osteogenic differentiation through PIK3CA inhibition. Additionally, *S. aureus* upregulates DAPK3, promoting osteoblast autophagy via the AMPK/ULK1 pathway while inhibiting the mTOR/GPX4 axis. It promotes CHI3L1 transcription by suppressing miR‐24, and CHI3L1 induces osteoblast apoptosis through p38MAPK and SMAD signaling. SpA directly binds to TNFR1 on osteoblasts, activating NF‐κB and JNK pathways to induce apoptosis and IL‐6 secretion. Hypoxia following *S. aureus* infection activates HIF‐1α, inducing STUB1 expression and leading to osteogenic defects and exacerbated inflammation.

Bone homeostasis refers to the collaboration between osteoblasts and osteoclasts, responsible for the formation and resorption of the bone extracellular matrix. With the immune evasion and persistent colonization of *S. aureus*, bone homeostasis is disrupted under the combined effects of *S. aureus* and various immune cells [204, 205]. Upon contact with or internalization of *S. aureus*, the first line of defense by osteoblasts is the secretion of inflammatory factors, such as cytokines, chemokines, and growth factors, which can activate and recruit immune cells from both the innate and adaptive immune systems [206]. The close relationship between osteoclasts and osteoblasts is also modulated by *S. aureus* infection [103]. Osteoclasts are the only cell type with bone resorption function, its overactivation is closely related to excessive bone loss (Figure 4) [207]. Disruption of bone balance in OM is essentially driven by a series of abnormally activated or inhibited cell signaling pathways. These pathways form the molecular bridge connecting bacterial infections to bone homeostasis imbalances (Figure 5) [91, 208].

#### 6.3.1 *S. aureus* Promotes Osteoclastogenesis

Preliminary studies show that *S. aureus* induces osteoclastogenesis via NF‐κB signaling activated by its cell wall components and secreted soluble molecules [209]. SpA, a key cell wall virulence factor [210], activates NF‐κB and MAPK pathways through TNFR1 and EGFR to drive osteoclast formation [211, 212, 213], and NF‐κB inhibition with JSH‐23 suppresses this effect [209, 211]. *S. aureus* also drives osteoclast generation via IL‐1R/MyD88 signaling [214]. Additionally, it activates the NLRP3 inflammasome through NF‐κB, promoting osteoclast‐specific gene expression and bone resorption [215], an effect abolished by MCC950 pretreatment, indicating that both NLRP3 and NF‐κB pathways mediate *S. aureus*‐induced osteoclast differentiation and bone resorption. Chitinase 3‐like 1 (CHI3L1), a member of the GH18 family involved in vascular cell adhesion and migration [216], is significantly upregulated in OM patients and mice [217]. CHI3L1 expression rises early during osteoclast differentiation, and its siRNA silencing impairs bone resorption [218]. Mechanistically, CHI3L1 binds to IL‐13Rα2 to enhance RANKL‐induced MAPK and AKT activation, promoting osteoclast differentiation [219]. *S. aureus* peptidoglycan (PGN‐sa), a major cell wall component and key pathogenic factor in OM [220], promotes osteoclast formation via TLR2‐mediated NF‐κB/NFATc1 signaling, thereby facilitating OM progression [221].

#### 6.3.2 *S. aureus* Inhibits Osteogenic Differentiation

Soluble factors derived from *S. aureus* biofilms drive bone loss in chronic OM by impairing osteoblast survival and osteogenic differentiation, thereby limiting new bone formation, while simultaneously promoting bone resorption through RANKL upregulation in osteoblasts (Figure 5) [222, 223]. SpA further enhances TLR2 expression in osteoblasts via the TNFR1/JNK pathway [224], leading to increased RANKL production and subsequent osteoclastogenesis and bone resorption [224, 225]. Moreover, SpA binding to osteoblasts suppresses key osteogenic markers (alkaline phosphatase, Type I collagen, osteopontin, and osteocalcin) thereby inhibiting new bone formation [224]. At the epigenetic level, SpA downregulates SIRT1, a highly conserved deacetylase involved in regulating apoptosis, lipid metabolism, oxidative stress, and inflammation [226, 227]. Conversely, SIRT1 overexpression promotes bone formation by enhancing FOXO1/3 phosphorylation and activating FOXO signaling [228]. USP14, the sole deubiquitinating enzyme on the 19S proteasome within the UPS, stabilizes SIRT1 through deubiquitination, which in turn promotes osteogenic differentiation markers such as RUNX2 and OCN [229]. Notably, both USP14 and SIRT1 are downregulated in OM bone tissues [229]. Additionally, SpA induces PMEPA1 upregulation in hBMSCs to inhibit osteogenic differentiation via the p38MAPK/NLRP3 axis [230], and upregulates METTL3, whose m6A modification drives miR‐320a expression to suppress osteogenic differentiation through PIK3CA inhibition [231]. Collectively, these findings highlight that SpA orchestrates bone loss through multifaceted mechanisms spanning receptor signaling, epigenetic regulation, and post‐translational modifications.

#### 6.3.3 *S. aureus* Promotes Osteoblast Apoptosis and Inflammation

*S. aureus* activates NF‐κB in osteoblasts, inducing IL‐6, IL‐12, and MCP‐1 secretion [232, 233]. CHI3L1 is upregulated in infected mouse femurs, and its inhibition alleviates OM pathology [217]; CHI3L1 promotes osteoblast apoptosis via p38/MAPK and SMAD signaling, exacerbating OM [234]. miR‐24, downregulated in OM and infected cells, directly targets CHI3L1 to counteract these effects [235]. SpA binds osteoblast TNFR1 to activate NF‐κB and JNK, inducing apoptosis and IL‐6 secretion [236, 237, 238, 239]. In OM and infected cells, TNF‐α and miR‐129‐5p are upregulated while eNOS is downregulated, with miR‐129‐5p directly targeting eNOS 3′‐UTR [240]. Postinfection, hypoxia activates HIF‐1α and induces STUB1, leading to osteogenesis defects and inflammation [241]. Additionally, *S. aureus* upregulates DAPK3 in BMSCs, modulating AMPK/ULK1 and mTOR/GPX4 to inhibit osteoblast autophagy [242]. Collectively, these findings suggest that *S. aureus* orchestrates a complex regulatory network encompassing inflammatory signaling, noncoding RNA interference, hypoxic stress, and autophagic dysfunction, highlighting potential multitarget therapeutic strategies for OM intervention.

#### 6.3.4 Immune Regulations of Bone Homeostasis

**FIGURE 6:**
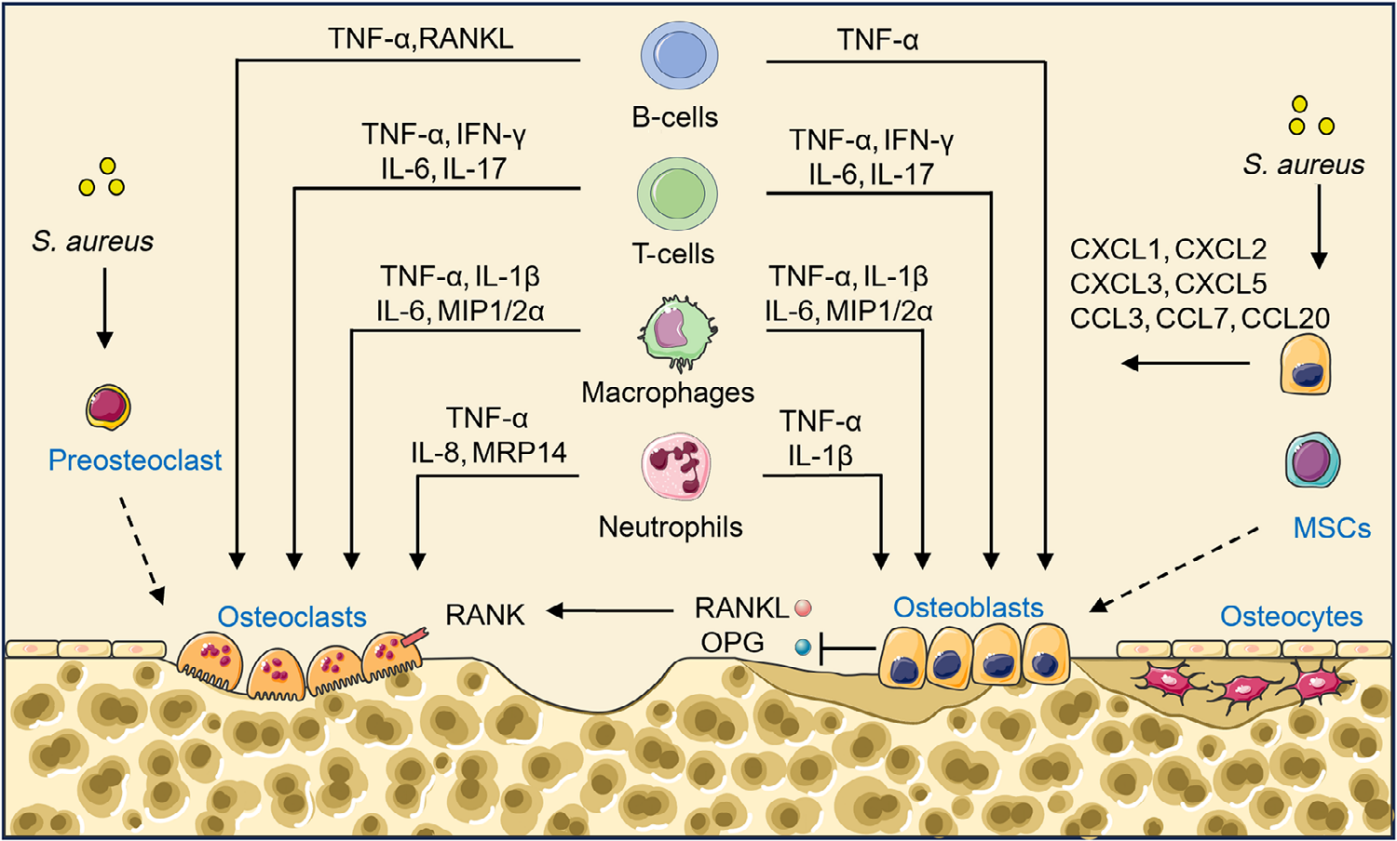
Immunoregulation of bone homeostasis. After invasion by *S. aureus*, a robust innate and adaptive immune response is triggered, further regulating bone homeostasis. Upon *S. aureus* infection, osteoblasts recruit immune cells by producing chemokines (CXCL1, CXCL2, CXCL3, CXCL5, CCL3, CCL7, and CCL20) and proinflammatory cytokines. Immune cells, including neutrophils, macrophages, T cells, and B cells, secrete proinflammatory factors at the infection site, which not only inhibit osteoblast differentiation and OPG expression but also promote RANKL expression in osteoblasts, thereby exacerbating inflammatory bone loss. Macrophages stimulated by *S. aureus* secrete TNF‐α, IL‐1β, IL‐6, and MIP1/2α to stimulate osteoclast precursor differentiation and maturation. Neutrophils promote osteoclastogenesis through TNF‐α, IL‐8, and MRP‐14. T‐cell‐derived proinflammatory cytokines (TNF‐α, IFN‐γ, IL‐6, and IL‐17) directly promote osteoclastogenesis while inducing osteoblasts to express more RANKL and proinflammatory factors, further recruiting and activating osteoclasts. B cells promote osteoclastogenesis by secreting RANKL and inflammatory factors such as TNF‐α.

*S. aureus* invasion triggers a robust innate and adaptive immune response, yet its potent immune evasion and persistence drive chronic inflammation. This persistent inflammatory milieu remodels immune and bone cells within the microenvironment, severely disrupting bone homeostasis (Figure 6) [243, 244]. Osteoblast differentiation is governed by multiple signals, with immune‐derived components serving as integral regulators [245]. *S. aureus* upregulates G‐CSF in OM patients to suppress osteoblast function and induce progressive bone loss [246]. G‐CSF further promotes IL‐1β expression in neutrophils, which inhibits osteoblast differentiation via the JNK/P53 and JNK/BCL2 pathways [247]. Immune dysregulation in OM triggers an inflammatory cascade that disrupts the osteoblast–osteoclast balance [205]. Infected osteoblasts produce proinflammatory cytokines and chemokines (CXCL1, CXCL2, CXCL3, CXCL5, CCL3, CCL7, CCL20) to recruit immune cells [170, 248, 249, 250], while proinflammatory cytokines simultaneously inhibit osteoblast differentiation and OPG expression while promoting RANKL expression, thereby driving inflammatory bone loss [246, 251, 252].

The macrophage–osteoclast axis is central to osteoimmunology [253]. Upon *S. aureus* stimulation, macrophages release abundant proinflammatory cytokines (TNF‐α, IL‐1β, IL‐6, MIP1/2α), directly stimulating osteoclast precursor differentiation and enhancing mature osteoclast resorption activity [254, 255, 256]. *S. aureus* infection induces osteoclast precursors to differentiate into activated macrophages that actively secrete proinflammatory cytokines, further enhancing resorption and promoting osteoclast generation at infection sites [257]. Chemokine upregulation recruits neutrophils that secrete TNF‐α, IL‐8, and MRP‐14 to promote osteoclastogenesis [258, 259]. T‐cell‐derived cytokines (TNF‐α, IFN‐γ, IL‐6, IL‐17) directly drive osteoclast production while inducing osteoblasts to express more RANKL and proinflammatory factors, further recruiting and activating osteoclasts [205, 260]. Recent studies also show elevated RANKL‐dependent osteoclastogenesis with increased B‐cell levels and heightened TNF‐α secretion in OM [259]. These findings underscore that bone destruction in OM is not a direct consequence of bacterial infection per se, but rather a result of complex immune–bone crosstalk that sustains a vicious cycle of inflammation and resorption. This perspective shifts the therapeutic paradigm from purely antibacterial strategies toward a comprehensive approach integrating antimicrobial therapy, immunomodulation, and bone metabolism management to effectively halt OM progression.

## 7 Management

The management of OM remains challenging due to the complex interplay among persistent infection, immune dysregulation, and bone metabolic imbalance. This section provides an overview of current therapeutic approaches and emerging targeted strategies. We first discuss traditional treatments (antibiotics and surgical debridement), highlighting their limitations in achieving durable remission amid rising antibiotic resistance and the refractory nature of the disease. We then shift to potential targeted therapeutic strategies, including eradicating *S. aureus* through novel antipathogen agents, restoring immune homeostasis by modulating key immune cell populations, and rebalancing bone metabolism via regulation of osteoclast and osteoblast activities.

### 7.1 Traditional Treatment

Currently, no consensus exists on the clinical classification and treatment of OM. Conventional therapy relies on antibiotics plus surgical debridement (Table 1) [38, 52]. However the prolonged course burdens patients with pain and discomfort, demands extensive clinician effort, and incurs high costs for families and society [33]. Despite surgical and chemotherapeutic advances, progress remains limited; the refractory and complex nature of OM often leads to treatment failure and recurrence [22, 261]. Anti‐infective therapy remains the mainstay, yet rising bacterial resistance is undermining traditional antibiotics, driving interest in novel agents [262, 263]. Treatment success hinges on early diagnosis, targeted and sustained anti‐infective therapy, and debridement/necrotic area management, requiring multidisciplinary collaboration [264, 265]. Nevertheless, achieving complete eradication of the disease remains a major challenge. Further elucidating the pathogenic mechanisms is therefore critical for optimizing treatment, improving cure rates, and enhancing patient quality of life.

**TABLE 1:** Ongoing clinical trials on the treatment of OM.

Type of treatment	Drug/therapy	Status	Phases	Objectives	References
Intravenous antibiotics	Zoledronate	Not yet recruiting	II	Evaluate the efficacy of zoledronate in NSAID‐resistant chronic recurrent multifocal OM	NCT07668583
Intravenous or oral antibiotics	Amoxicillin‐clavulanate, clindamycin, levofloxacin	Not yet recruiting	IV	Compare the efficacy of oral and IV antibiotics in the treatment of orofacial OM	NCT05867654
Oral antibiotics	β‐Lactam antibiotics, non‐β‐lactam antibiotics	Recruiting	IV	Compare different classes of oral antibiotics (β‐lactam and non‐β‐lactam antibiotics) for the treatment of bone infections of the leg and foot	NCT07266753
	Trimethoprim sulfamethoxazole	Recruiting	IV	Evaluate trimethoprim sulfamethoxazole works to treat invasive infections due to methicillin‐resistant *S. aureus* in children	NCT06982105
Intravenous and oral antibiotics	Bisphosphonates combined with vancomycin	Not yet recruiting	III	Evaluate the efficacy and safety of bisphosphonates combined with vancomycin in the treatment of refractory OM	NCT07435051
Local antibiotics injection	Antibiotic‐loaded calcium sulfate beads	Not yet recruiting	IV	Evaluate the effectiveness of absorbable antibiotic beads in treating lower extremity infections and compare it to the current standard care.	NCT07072923
Debridement combined with local antibiotics	Cerament V/G	Not yet recruiting	Not applicable	Evaluate the clinical and radiological healing response of bone infection following treatment with the antibiotic paste Cerament V/G	NCT06628115
	Cerament‐G, gentamicin	Recruiting	Not applicable	Evaluate the Efficiency of the Bone Substitute Cerament‐G Locally Delivering Gentamicin in the Treatment of Chronic OM of Long Bones	NCT04805164
	Genex with gentamicin	Recruiting	Not applicable	Evaluate genex with gentamicin an antibiotic‐loaded bone void filler, works to treat and is a safe option as part of the surgical treatment of OM in the extremities	NCT07270380
Hyperbaric oxygen	Hyperbaric oxygen therapy	Recruiting	Not applicable	Evaluates the effect of additional hyperbaric oxygen therapy after lower extremity amputation because of OM	NCT03594344
Local antibiotic prophylaxis	Vancomycin hydrochloride, gentamicin	Recruiting	III	Evaluate if treating tibia fracture patients with a calcium sulfate antibiotic depot is better at preventing infection that the standard of care	NCT05766670
	Antibiotic‐coated intramedullary nail	Recruiting	IV	Compare outcomes between patients treated primarily with a prophylactic antibiotic‐coated nail and those treated with traditional standard of care intramedullary nailing	NCT05421741

*Data Source*: ClinicalTrials.gov.

#### 7.1.1 Antibiotics

Antibiotic selection should be individualized based on patient susceptibility. Traditional therapies such as vancomycin and clindamycin remain effective for acute hematogenous OM. When these are inadequately effective, linezolid, and ceftolozane, and long‐acting lipoglycopeptides also show early promise [266]. Zoledronate has shown efficacy in nonsteroidal anti‐inflammatory drug‐resistant recurrent multifocal OM (NCT07668583). The combination of rifampin with other antibiotics can improve cure rates, particularly in cases involving pseudarthrosis or spinal implant infections [267, 268]. Oral antibiotics have efficacy and safety comparable to intravenous therapy (NCT05867654) [39, 269]. Oral treatment avoids the risks associated with intravenous catheters and incurs lower costs, making it a reasonable choice for OM caused by susceptible organisms [270]. Oral regimens typically last 4–6 weeks, although the optimal duration has not been fully established [271]. Therapeutic efficacy may vary among oral antibiotic classes (β‑lactam vs. non‑β‑lactam) in the treatment of lower‑extremity bone infections (NCT07266753). Oral trimethoprim‐sulfamethoxazole may be effective against invasive methicillin‐resistant *S. aureus* infections in pediatric patients (NCT06982105). Oral trimethoprim‐sulfamethoxazole plus ciprofloxacin plus amoxicillin achieves an 81% response rate [31]. Oral bisphosphonates combined with intravenous vancomycin represent a promising strategy for refractory OM (NCT07435051). Additionally, local therapy may be achieved via the injection of antibiotic‑impregnated absorbable calcium sulfate beads into the site of infection (NCT07072923). For chronic OM, there is no evidence supporting longer antibiotic courses over shorter ones; given the risk of resistance, determining the optimal antibiotic regimen and duration, as well as the role of surgical debridement, remains an important and unmet clinical need.

#### 7.1.2 Debridement

Chronic OM responds poorly to nonsurgical treatment, primarily due to biofilm‐protected pathogens within the lesion. These pathogens attach to necrotic bone or implants, forming sanctuaries that evade immune clearance and antibiotics. Thus, curative treatment hinges on surgical removal of biofilm colonies, supplemented by antibiotics to eliminate residual planktonic phenotypes, alongside optimization of host immunity and systemic status [267]. Surgical debridement and drainage of soft tissue abscesses remain the cornerstone, though consensus on which cases require debridement is lacking [272]. In practice, debridement is typically part of initial treatment, especially with internal fixation or necrotic bone, and skeletal stabilization is essential to shorten healing and reduce complications. Debridement with antibiotics shortens hospital stays, lowers costs, ensures better infection control, and avoids adverse effects of prolonged systemic therapy [273]. Following surgical sequestration removal and purulent drainage, antibiotic‑loaded bone substitutes (such as Cerament V/G or Genex with gentamicin) may be directly implanted into the bony defect to provide effective local antibiotic therapy (NCT06628115, NCT04805164, NCT07270380). Antibiotic‐impregnated collagen sponges may be used during surgery; some studies suggest improved local control, but evidence quality is low and bias risk high, warranting caution [274]. Hyperbaric oxygen therapy may be adjunctive in select cases, particularly with ischemia or poor healing, though its efficacy needs further validation (NCT03594344) [275]. For severe open tibial fractures, local antibiotic prophylaxis can be achieved at the time of definitive intramedullary fixation using calcium sulfate antibiotic reservoirs or antibiotic‑coated intramedullary nails (NCT05766670, NCT05421741).

### 7.2 Potential Targeted Therapeutic Strategies

OM is a complex disease with an imbalance in the interaction of infection, immunity, and bone metabolism, and successful treatment requires comprehensive intervention to target pathogens, abnormal immune responses, and bone metabolism imbalances. *S. aureus* uses a series of toxins and virulence proteins to kill and replace immune cells during infection, producing non‐neutralizing disease‐causing antibodies to achieve immune escape. For *S. aureus* protein, which may provide new targets for antibiotics and immunotherapies. At the same time, based on the understanding of the above pathogenesis, targeted therapy for the immune system provides new hope for OM, especially refractory chronic OM (Table 2).

**TABLE 2:** Potential targeted therapy for OM.

Target	Agent	Mechanisms	References
*S. aureus*	*B. subtilis* CFS	Inhibiting the secretion of adhesion molecules and virulence factors, as well as biofilm formation in *S. aureus*	[276]
	I‐A	Binding to ClpP and downregulating its activity to reduce bacterial adhesion	[280]
	Anti‐GMD	Suppressing staphylococcal abscess communities and reducing bacterial load	[281]
	Anti‐SpA	Blocking SpA‐mediated inhibition of osteoblast activity and induction of osteoclast formation	[282]
	Antibody‐PSA conjugate	Inducing surface calcification to block metabolism, while stimulating macrophage activation and S100A8/S100A9 expression	[283]
Neutrophils	IL‐27	Inducing the accumulation of proinflammatory neutrophils to reduce abscess formation, and inhibiting RANK expression and osteoclast differentiation	[286]
	HBD3	Promoting NADPH oxidase‐dependent ROS production and NET release by activating the Mrgpra2 downstream signaling pathway	[119]
Macrophages	STM2457	Inhibiting the production of IL‐6 and TNF‐α by downregulating the TLR4/MyD88/NF‐κB pathway in macrophages	[287]
	Mn^2^^+^	Repressing Sirt3 and inhibiting PINK1/parkin‐mediated mitophagy, leading to mtROS accumulation	[288]
MDSCs	mAb1A8	Targeted depletion of Ly6G^+^ MDSCs.	[155]
	TAS4464	Inhibiting the expansion of MDSCs and M2 macrophages by binding to NEDD8 to suppress neddylation	[163]
	GMI	Suppressing the immunosuppressive effects of MDSCs	[292]
	CURN	Suppressing the expansion of biofilm‐induced MDSCs	[296]
	Celecoxib	Inhibiting the expansion of COX‐2‐induced MDSCs	[299]
Tregs	WWXDD	Inhibiting the activation of the IL‐2/STAT5 signaling pathway and the expression of TAZ to suppress the development and function of Treg cells.	[302, 303]
	CGA	Inhibiting IL‐2‐mediated expansion of Tregs	[305]
B cells	ASCs	Inhibiting the abnormal proliferation of B cells and RANKL expression Th17 cells differentiation	[307]
Osteoclasts	Denosumab	inhibit osteoclast‐mediated bone resorption by binding to RANKL	[309]
Osteoblasts	ISO‐1	Reversing SpA‐mediated inhibition of osteogenic differentiation by inhibiting the NF‐κB signaling pathway	[239]
	IDF‐11774	Inhibits the expression of inflammatory factors in osteoblasts by suppressing the HIF‐1α/STUB1 signaling pathway	[241]
	ISL and vancomycin	Suppressing the inflammatory response induced by *S. aureus* by inhibiting the NF‐κB and MAPK signaling pathways	[313]
	Butyrate	Inhibiting the upregulated expression of METTL3 induced by *S. aureus*, and promotes the expression of osteogenesis‐related genes	[314]
	Naringin	Promoting osteogenesis by upregulating Notch signaling in BMSCs.	[317]
	Azithromycin and kaempferol	Suppressing the expression of inflammatory factors by inhibiting oxidative stress and the phosphorylation of ERK1/2 and SAPK	[318]
	Artesunate	Attenuating inflammation and promoted osteogenic differentiation by inhibiting the NOD2/p65 pathway	[319]
	CTLA4‐Ig	Promoting osteoblast differentiation by activating the Wnt/β‐catenin signaling pathway and modulates the inflammatory microenvironment	[320]

Abbreviations: ASCs, adipose‐derived stem cells; *B. subtilis* CFS, *Bacillus subtilis* cell‐free supernatant; CGA, chlorogenic acid; CURN, curcumin nanoparticle; GMD, glucosaminidase; GMI, *Ganoderma microsporum* immunomodulatory protein; HBD3, latest research has revealed that human β‐defensin 3; I‐A, isochlorogenic acid A; ISL, isoliquiritigenin; SpA, staphylococcal protein A; WWXDD, Wuwei Xiaodu Drink.

#### 7.2.1 Targeting *S. aureus*

Potential targeted therapeutic strategies against *S. aureus* OM are being explored from multiple dimensions, including the use of microbial products, virulence factor inhibition, and combined immunotherapy. *Bacillus subtilis* cell‐free supernatant (CFS) not only kills planktonic bacteria and biofilms but also significantly increases bacterial susceptibility to penicillin and gentamicin by inhibiting adhesion molecules and virulence factor secretion while improving membrane permeability; in animal models, it effectively enhances penicillin efficacy, and its active components are derived from bacterial peptides, positioning it as a potential resistance modifier for β‐lactam antibiotics [276, 277]. In terms of virulence factor targeting, the polyphenol derivative isochlorogenic acid A (I‐A) has been identified as an efficient inhibitor of ClpP, a key virulence regulator in *S. aureus* that coordinates drug resistance, regulates adhesion, and acts on numerous virulence targets [210, 211]. I‐A downregulates ClpP activity and reduces bacterial stress responses, with demonstrated in vivo efficacy in rat OM models, thereby offering new insights into virulence‐directed therapeutic approaches [280]. Additionally, combination therapy targeting the cell wall synthesis enzyme GMD with anti‐GMD antibodies and vancomycin has shown synergistic effects in PJI models, significantly inhibiting infection, reducing osteolysis, and promoting new bone formation, highlighting the advantage of integrating immunotherapy with antibiotics [191, 192, 281]. Furthermore, anti‐SpA antibody therapy, which neutralizes SpA to block immune evasion and osteoclast activation, not only reduces bone‐degrading enzyme expression and trabecular bone loss but also serves as an adjunctive antivirulence strategy to prevent bacterial escape from antimicrobials and the immune system [282], collectively laying a solid foundation for comprehensive management of OM. A recent study reported that an antibody‐PSA conjugate eradicates MRSA by inducing surface calcification to block metabolism, while stimulating macrophage activation and S100A8/S100A9 expression to enhance immune clearance, showing high efficacy and safety in murine chronic OM models [283].

#### 7.2.2 Targeting the Immune Microenvironment

##### 7.2.2.1 Targeting Neutrophils

IL‐27 is a heterodimeric cytokine belonging to the IL‐12 cytokine family, which is mainly produced by antigen‐presenting cells such as macrophages, monocytes, and dendritic cells [284, 285]. The study found that *S. aureus* induces IL‐27 expression in osteoblasts and macrophages in early OM through TLR activation [286]. IL‐27 secretion relies on the self‐regulated IL‐27 signaling pathway. However, endogenous IL‐27 does not affect host susceptibility to OM. And by administering rAAV‐IL‐27p28 at supraphysiological levels to express IL‐27, it induces the accumulation of proinflammatory neutrophils, thereby reducing abscess formation and enhancing bacterial clearance at the site of infection [286]. In addition, the abundance of IL‐27 inhibits RANK expression and inhibits osteoclast differentiation, leading to reduced osteolysis during chronic OM [286]. Latest research has revealed that human β‐defensin 3 (HBD3) promotes NADPH oxidase‐dependent ROS production and NET release by activating the Mrgpra2 downstream signaling pathway in synergy with the TNFα/TNFR2 signaling pathway [119]. This indicates that the defensin–Mrgpra2 axis serves as a key regulatory hub, enhancing pathogen clearance while mitigating tissue damage caused by uncontrolled inflammation or neutrophil exhaustion. This provides a highly promising therapeutic strategy for infectious bone diseases such as OM.

##### 7.2.2.2 Targeting Macrophages

STM2457 is an inhibitor of METTL3. Isolated BMDMs from OM patients inhibited LPS‐induced IL‐6 and TNF‐α secretion after STM2457 pretreatment [287]. In *S. aureus*‐induced OM C57BL/6 mouse model, STM2457 alleviated reactive bone formation weakening and cortical bone loss [287]. In addition, STM2457 pretreatment downregulated the relative expressions of MyD88, p‐TAK and p‐IKKα/β in LPS‐stimulated BMDMs [287]. It indicates that the protective effect of STM2457 on OM mice is achieved by downregulating the TLR4/MyD88/NF‐κB pathway in macrophages. Recent studies show that Mn^2^^+^ eradicates intracellular *S. aureus* by repressing Sirt3 and inhibiting PINK1/parkin‐mediated mitophagy, leading to mtROS accumulation [288]. This highlights mitochondrial quality control as a novel therapeutic target against intracellular infection.

##### 7.2.2.3 Targeting MDSCs

Immunomodulatory strategies targeting MDSCs and the inflammatory microenvironment have also shown therapeutic promise for *S. aureus* OM. mAb1A8, a Ly6G‐specific monoclonal antibody, depletes Ly6G^+^ cells [289, 290]. Given that MDSCs promote biofilm chronicity by suppressing monocyte/macrophage proinflammatory activity, mAb1A8‐mediated clearance enhances this activity and significantly improves bacterial and biofilm clearance [155]. Biofilms exploit the upstream neddylation pathway to drive the expansion of MDSCs and M2 macrophages [163]. The NEDD8‐activating enzyme inhibitor TAS4464 effectively suppresses this expansion and attenuates bone destruction and inflammation in PJI models [163]. Natural products also show potential. *Ganoderma microsporum* immunomodulatory protein (GMI), a fungal immunomodulatory peptide from *Ganoderma lucidum* [291], inhibits MDSC accumulation and promotes cytotoxic T‐cell proliferation by inducing IL‐6/TNF‐α and suppressing ROS in MDSCs [292]. Curcumin nanoparticles (CURNs) overcome curcumin's poor bioavailability [293, 294, 295], and reverse biofilm‐induced MDSC‐mediated T‐cell suppression in PJI models [296]. Additionally, the COX‐2 inhibitor celecoxib counteracts COX‐2 upregulation in MDSCs, neutrophils, and macrophages during OM [297, 298, 299], inhibiting inflammatory gene expression and rescuing bone loss, thus offering therapeutic potential for *S. aureus*‐induced bone destruction [299]. These immunomodulatory approaches complement direct antivirulence strategies and may enhance combination therapy outcomes.

##### 7.2.2.4 Targeting Treg Cells

Tregs undergo aberrant differentiation and expansion in *S. aureus* OM, mediating immune escape and promoting disease progression, making them a potential therapeutic target. Wuwei Xiaodu Drink (WWXDD), a traditional Chinese medicine prescription composed of honeysuckle, wild chrysanthemum, dandelion, *Viola philippica*, and sunflower, is widely used for heat‐clearing, detoxification, and treating skin infections [300, 301], and is also prescribed for OM in clinical practice. Studies have shown that WWXDD inhibits aberrant Treg differentiation and function both in vitro and in vivo, thereby suppressing OM progression [302, 303]. Mechanistically, it mainly targets the IL‐2/STAT5 signaling pathway and the transcriptional coactivator TAZ to interfere with Treg development and immunosuppressive function, reducing chronic inflammation and preventing OM progression toward bone tumors [302, 303]. Chlorogenic acid (CGA), a plant‐derived secondary metabolite with diverse pharmacological activities [304], has also shown therapeutic potential. In post‐traumatic OM animal models, Treg proportion increases in peripheral blood, accompanied by elevated Foxp3, Ctla‐4, IL‐10, and IL‐2 expression and reduced TNF‐α levels; intravenous IL‐2 further expands Tregs and exacerbates bone destruction [305]. In contrast, CGA treatment reduces Treg proportion and downregulates Foxp3, Ctla‐4, and IL‐10 expression [305], indicating that CGA alleviates OM by inhibiting IL‐2‐mediated Treg upregulation. Collectively, these findings suggest that Treg‐targeted immunomodulation represents a promising avenue for OM therapy.

##### 7.2.2.5 Targeting B Cells

Adipose‐derived stem cells (ASCs) hold great promise in improving bone healing due to their superior isolation procedures, growth kinetics, plasticity, and nutritional properties [306]. OM exhibits decreased osteoblastogenesis and increased osteoclastogenesis, indicating elevated bone resorption accompanied by an increased secretion of RANKL from B cells [259]. In mouse animal models, debridement of infected bone followed by topical treatment with ASCs found that ASCs can restore bone healing by increasing osteoblast production and downregulating osteoclasts [307]. Mechanistically, ASCs regulate bone homeostasis in OM by inhibiting the abnormal proliferation of B cells and RANKL expression [307]. The complex interaction mechanism of stem cells in bone infection and immunity has been revealed.

#### 7.2.3 Targeting Bone Homeostasis

##### 7.2.3.1 Targeting Osteoclast

Denosumab is a human monoclonal antibody that acts as a novel drug to inhibit osteoclast‐mediated bone resorption by binding to RANKL produced by osteoblasts [308]. OM *S. aureus* infection potently stimulates RANKL expression in bone marrow mesenchymal stem cells. Mice were treated with Denosumab both before and during infection. Although the bacterial load within the femur is not affected, the use of Denosumab completely avoids severe cortical bone destruction caused by infection [309]. In addition, In Denosumab‐treated mice, osteoclasts were significantly reduced near the seeding site [309]. These results suggest that RANKL‐mediated osteoclast formation is a significant factor in the development of *S. aureus* infection is necessary for bone loss. Treatment with Denosumab improves OM by inhibiting the formation of osteoclasts.

##### 7.2.3.2 Targeting Osteoblast

Multiple strategies have been explored to protect osteoblasts from *S. aureus*‐induced dysfunction and promote bone regeneration in OM. ISO‐1, an MIF inhibitor [310], reverses SpA‐mediated inhibition of osteogenic differentiation via NF‐κB suppression, counteracting MIF expression in BMSCs that impairs bone formation [239]. IDF‐11774, a hypoxia inhibitor [311, 312], targets the upregulated HIF‐1α/STUB1 axis in infected osteoblasts; it enhances osteogenic markers (OSX, RUNX2, ALP) and reduces inflammatory cytokines (IL‐6, IL‐1β, CRP), alleviating bone destruction in OM models [241]. The combination of isoliquiritigenin (ISL) and vancomycin shows synergistic efficacy in rat OM models, reducing infection severity and bacterial burden versus monotherapy [313]. ISL attenuates inflammation via NF‐κB/MAPK inhibition while promoting osteogenesis through BMP/SMAD signaling and Runx2/BMP2/ALP upregulation [313]. Butyrate counteracts *S. aureus*‐induced METTL3 upregulation in osteoblasts, rescuing osteogenic markers and autophagy balance [314]. In vivo, it alleviates inflammation and restores osteogenic activity in implantation‐associated OM, suggesting its therapeutic potential [314]. Naringin, a flavonoid with bone‐healing effects [315, 316], alleviates cortical bone loss in OM by promoting osteogenesis via Notch signaling in BMSCs [317]. The combination of azithromycin and kaempferol exhibits enhanced antibacterial and antibiofilm effects while reducing osteoblast oxidative stress and ERK1/2/SAPK phosphorylation, decreasing inflammation and bone destruction; kaempferol may increase azithromycin bioavailability by inhibiting biofilms [318]. Artesunate attenuated inflammation and promoted osteogenic differentiation in rat bone marrow MSCs by inhibiting the NOD2/p65 pathway [319]. In vivo, Artesunate facilitated the healing of chronic suppurative mandibular OM in rats [319]. Recent studies have reported that CTLA4‐Ig promotes osteoblast differentiation by activating the Wnt/β‐catenin signaling pathway and modulates the inflammatory microenvironment to preserve bone integrity, offering a promising dual‐acting therapeutic strategy for OM [320]. Collectively, these osteoblast‐targeted approaches offer multifaceted strategies to counteract infection‐induced bone loss and promote skeletal repair in OM.

#### 7.2.4 Challenges and Considerations for Immunotherapies

Despite the considerable promise of the aforementioned immunotherapeutic strategies, several challenges remain for their clinical translation. First, off‐target effects are a major concern, as immunomodulatory agents may inadvertently affect systemic immunity or disrupt normal bone homeostasis, leading to impaired wound healing or altered bone remodeling. Second, the long‐term risk of immunosuppression is a critical issue, particularly for chronic OM patients requiring prolonged therapy, as sustained immune modulation may predispose them to secondary infections or malignancies, necessitating close monitoring and defined treatment duration. Third, combination therapies, while synergistic, may introduce unforeseen adverse effects; concurrent use of immunomodulators with antibiotics requires rigorous pharmacokinetic and pharmacodynamic evaluation to avoid antagonism or enhanced toxicity. Fourth, the optimal timing and dosing of immunotherapies remain poorly defined, as patients' immune status varies across disease stages, and inappropriate intervention could exacerbate inflammation or prove ineffective. Additionally, patient heterogeneity in age, comorbidities, and genetic background may influence treatment responses and complicate generalization of trial results. Finally, the high cost and manufacturing complexity of biologics pose significant economic and logistical barriers to widespread clinical adoption. Addressing these challenges will require well‐designed preclinical and clinical studies, along with the development of predictive biomarkers to guide patient selection and monitor treatment responses, ultimately ensuring that immunotherapies for OM are both safe and effective.

## 8 Conclusion and Prospects

In recent years, the spotlight has increasingly turned to the role of immune cells in OM, revealing the immune system's intricate and multifaceted involvement in the disease. From the perspective of pathogenesis, a well‐regulated and appropriately controlled immune response is crucial for clearing the infection, whereas an impaired immune response not only fails to contain the pathogen but also exacerbates infection, ultimately driving bone destruction and chronic disease progression. During the chronic phase of OM, immune cell function is governed by a complex interplay of inflammatory cytokines, immune checkpoints, metabolic alterations, and transcription factors, which collectively modulate the ability of immune cells to combat infection and maintain tissue homeostasis. Furthermore, immune cells are inextricably linked to the bone‐remodeling process through close interactions with osteoblasts and osteoclasts. A particularly concerning aspect of OM is the formation of a self‐reinforcing vicious cycle, in which local immunosuppression, persistent systemic inflammation, and ongoing bone destruction create a feedback loop that perpetuates the disease. This cycle underlies the prolonged clinical course of OM and its resistance to conventional therapies.

Despite significant progress in understanding the immune‐related aspects of OM, several technical bottlenecks remain that hinder the translation of immunotherapies into clinical practice. First, the molecular mechanisms governing immune cell function in the OM microenvironment remain incompletely elucidated, particularly regarding the dynamic crosstalk between different immune subsets and their spatial‐temporal regulation within infected bone tissue. Second, the lack of reliable biomarkers for patient stratification and treatment response monitoring poses a major obstacle to precision immunotherapy. Third, the complexity and heterogeneity of OM, varying across pathogens, infection sites, and host immune status, make it difficult to establish standardized treatment protocols. Fourth, the potential off‐target effects of immunomodulatory agents on systemic immunity and normal bone homeostasis raise safety concerns that require careful evaluation. Finally, the majority of current evidence is derived from preclinical animal models, which may not fully recapitulate the human disease spectrum, highlighting the urgent need for well‐designed clinical trials to validate the efficacy and safety of these emerging strategies. Moreover, given that some OM pathogens utilize human‐specific virulence mechanisms, the establishment of humanized mouse models is of great significance for facilitating the translation of preclinical research into clinical applications.

It should be acknowledged that this review focuses exclusively on *S. aureus*, which, although the predominant pathogen in OM, is not the sole causative agent. Other bacteria account for a substantial proportion of OM cases, particularly in polymicrobial or healthcare‐associated settings. These pathogens employ distinct immune evasion strategies, such as biofilm formation, toxin secretion, and modulation of host inflammatory responses, that differ considerably from those of *S. aureus*. Consequently, the immunotherapeutic approaches discussed herein may not be universally applicable across all OM etiologies. Future studies should investigate pathogen‐specific immune signatures and tailor immunomodulatory strategies accordingly, while also considering the potential for combination therapies that address the unique challenges posed by non‐*S. aureus* pathogens. This broader perspective will be essential for developing comprehensive treatment paradigms that are effective across the full spectrum of OM.

Exploring the precise mechanisms by which immune cells function in OM holds immense promise for the development of novel immunotherapies. Future research endeavors should be directed toward several key areas. First, precision immunotyping is essential. By clarifying the specific immune characteristics of OM patients, taking into account factors such as the causative pathogen and the clinical stage of the disease, we can pave the way for individualized immunotherapy that tailors interventions to each patient's unique immune profile. Second, timing of interventions will be of critical importance. Understanding the dynamic changes in the immune response throughout the course of OM will enable us to identify optimal windows for therapeutic intervention, thereby maximizing efficacy and minimizing side effects. Third, combination treatment strategies represent a promising avenue for overcoming refractory OM. Integrating immunotargeted therapy with traditional modalities such as antibiotics, surgical debridement, and bone graft materials can create a multimodal comprehensive treatment plan that addresses infection control, bone repair, and immune regulation simultaneously. Fourth, advanced technologies such as single‐cell sequencing, spatial transcriptomics, and organoid models should be leveraged to dissect the cellular heterogeneity and intercellular communication networks within the OM niche, providing a deeper mechanistic understanding to guide rational drug design. Meanwhile, the application of emerging analytical tools such as machine learning is expected to accelerate the discovery of biomarkers and the assessment of antimicrobial efficacy.

In conclusion, targeting the immune system as a therapeutic strategy marks a significant paradigm shift in the treatment of OM. Although most immunotherapies are still in the preclinical or early clinical research stages, their potential is vast. Currently, multiple clinical trials of phage therapies and novel immunomodulatory agents are underway, offering new hope for the treatment of OM. Other innovative approaches in advanced preclinical development, such as antimicrobial‐coated implants and bone‐targeted antibiotics, also demonstrate promising application prospects. With continued research and development, these innovative approaches may revolutionize the clinical management of this challenging disease. Additionally, considering the close relationship between immune cells, bone‐forming osteoblasts, and bone‐resorbing osteoclasts, future research should also delve deeper into how immunotherapies can be designed to precisely regulate bone homeostasis while modulating the immune microenvironment, thus achieving a more comprehensive and effective treatment for OM. Bone regeneration in defects caused by OM also represents an important future research direction. The integration of multidisciplinary efforts, including immunology, microbiology, bone biology, and clinical orthopedics, will be essential to translate these promising findings into tangible clinical benefits for patients.

## Author Contributions

Qingqiang Lei, Liangbin Lin, and Hui Yu conceived the paper and wrote the manuscript. Peidong Pu, Jie Chen, Xianchao Su, Shubao Liu, and Yanli Tong prepared the figures and tables. All the authors contributed to the article and approved the submitted version.

## Funding

This work was supported by Science and Technology Support Program of Sichuan Province (2026NSFSC1765); National Natural Science Foundation of China (82402108, 82502196); China Postdoctoral Science Foundation under Grant Number (2025M772671); Sichuan Province Special Funding Project for Postdoctoral Fellows (TB2024041); Health Commission of Chengdu Medical Research Program (2024014 and 2024063); The Third People's Hospital of Chengdu Scientific Research Project (2023PI18).

## Ethics Statement

The authors have nothing to report.

## Conflicts of Interest

The authors declare no conflicts of interest.

## Data Availability

The authors have nothing to report.
